# Complete spatial characterisation of *N-*glycosylation upon striatal neuroinflammation in the rodent brain

**DOI:** 10.1186/s12974-021-02163-6

**Published:** 2021-05-16

**Authors:** Ana Lúcia Rebelo, Francesco Gubinelli, Pauline Roost, Caroline Jan, Emmanuel Brouillet, Nadja Van Camp, Richard R. Drake, Radka Saldova, Abhay Pandit

**Affiliations:** 1 CÚRAM SFI Research Centre for Medical Devices, National University of Ireland, Galway, Ireland; 2 CEA, CNRS, MIRCen, Laboratoire des Maladies Neurodégénératives, Université Paris-Saclay, Fontenay-aux-Roses, France; 3 Department of Cell and Molecular Pharmacology and Experimental Therapeutics, Medical University of South Carolina, Charleston, USA; 4 National Institute for Bioprocessing Research and Training (NIBRT), University College Dublin, Dublin, Ireland; 5 UCD School of Medicine, UCD Conway Institute of Biomolecular and Biomedical, Dublin, Ireland

**Keywords:** *N*-glycosylation, Protein glycosylation, Glycomics, Neuroinflammation, Striatum, LPS model, Liquid chromatography, MALDI-MSI

## Abstract

**Background:**

Neuroinflammation is an underlying pathology of all neurological conditions, the understanding of which is still being comprehended. A specific molecular pathway that has been overlooked in neuroinflammation is glycosylation (i.e., post-translational addition of glycans to the protein structure). *N-*glycosylation is a specific type of glycosylation with a cardinal role in the central nervous system (CNS), which is highlighted by congenital glycosylation diseases that result in neuropathological symptoms such as epilepsy and mental retardation. Changes in *N-*glycosylation can ultimately affect glycoproteins’ functions, which will have an impact on cell machinery. Therefore, characterisation of *N-*glycosylation alterations in a neuroinflammatory scenario can provide a potential target for future therapies.

**Methods:**

With that aim, the unilateral intrastriatal injection of lipopolysaccharide (LPS) in the adult rat brain was used as a model of neuroinflammation. In vivo and *post-mortem*, quantitative and spatial characterisation of both neuroinflammation and *N-*glycome was performed at 1-week post-injection of LPS. These aspects were investigated through a multifaceted approach based on positron emission tomography (PET), quantitative histology, reverse transcription-quantitative polymerase chain reaction (RT-qPCR), liquid chromatography and matrix-assisted laser desorption ionisation mass spectrometry imaging (MALDI-MSI).

**Results:**

In the brain region showing LPS-induced neuroinflammation, a significant decrease in the abundance of sialylated and core fucosylated structures was seen (approximately 7.5% and 8.5%, respectively), whereas oligomannose *N-*glycans were significantly increased (13.5%). This was confirmed by MALDI-MSI, which provided a high-resolution spatial distribution of *N-*glycans, allowing precise comparison between normal and diseased brain hemispheres.

**Conclusions:**

Together, our data show for the first time the complete profiling of *N-*glycomic changes in a well-characterised animal model of neuroinflammation. These data represent a pioneering step to identify critical targets that may modulate neuroinflammation in neurodegenerative diseases.

**Supplementary Information:**

The online version contains supplementary material available at 10.1186/s12974-021-02163-6.

## Background

Neuroinflammation is a complex pathology with multiple players that underlies most of the penetrating injuries (such as traumatic brain injury (TBI) and spinal cord injury (SCI)), and neurodegenerative conditions (such as Parkinson’s disease (PD), Alzheimer’s disease (AD), multiple sclerosis (MS) and amyotrophic lateral sclerosis (ALS) amongst others) [1–4]. Neuroinflammatory cascades are mainly governed by glial cells, specifically microglia, which are the resident macrophages (immune cells) of the CNS. These, together with astrocytes and neurons, can trigger the complement system and express related receptors, being part of the innate immune system [5–8]. Additionally, upon neuronal insults, microglia become activated and start producing neurotoxic pro-inflammatory mediators such as chemokines (C-C motif chemokine ligand 2 (CCL2) or C-X-C motif chemokine ligand 10 (CXCL10)), cytokines (e.g. interleukin 1β (IL-1β), interleukin 6 (IL-6), tumour necrosis factor α (TNF α), interferon-gamma (IFNγ)), reactive oxygen species (ROS), nitric oxide (NO), prostaglandins and other secondary messengers [9]. If the blood-brain barrier (BBB) is compromised, peripheral immune and endothelial cells can infiltrate the CNS, exacerbating the inflammatory cascades [10]. The duration and extent of neuroinflammation will dictate whether this will have a beneficial or detrimental outcome in the brain. Microglial activation is critical and necessary as a first-line in host defence due to phagocytosis and antigen presentation capability. However, chronic microglial activation can amplify inflammatory cascades, induce neuronal death and feed into a degenerative after-effect [10].

Lipopolysaccharide (LPS) is a potent immunostimulant that is naturally present in gram-negative bacteria’s cell wall, which binds predominantly to Toll-like receptor 4 (TLR-4) abundantly present in microglia [11, 12] and, to a lesser extent, in neurons and astrocytes [13–15]. Upon TLR-4 activation, different downstream pathways are triggered in microglia (including NF-kB and JAK-STAT cascades), ultimately leading to the activation of transcription factors and subsequent production of those above mentioned neurotoxic inflammatory factors [16, 17]. Administration of LPS in preclinical models induces, depending on the route of administration, behavioural impairments such as reduced locomotion, somnolence, memory deficiencies, decreased bodyweight, increased anxiety and general depression, amongst others, which are reminiscent of the clinical symptoms of neurodegenerative conditions like AD and PD [18]. Therefore, administration of LPS in rodents is well-established in the scientific community to model neuroinflammation-associated disorders (extensively reviewed by [18–20]). It has been widely used in mouse [21–23] and rat models [19, 24, 25], utilising distinct routes of administration, delivery methods, doses and timing of administration. Since its effect varies depending on the experimental design, experimental protocols are adapted to each study to achieve the optimal phenotype (acute vs chronic) with desired behavioural/cognitive outcomes. Moreover, LPS is also a powerful tool, both in vitro and in vivo, to further investigate the molecular and cellular mechanisms that take place upon neuroinflammation, and to test potential anti-inflammatory therapeutic strategies at the preclinical level [20].

A critical molecular aspect of neuroinflammation related to protein glycosylation has so far been overlooked. Proteins are fundamental players in any cell’s structure and functions, including a plethora of signalling cascades, cellular architecture, matrix organisation and biological interactions. Most proteins are post-translationally modified, with glycosylation being the most common of these modifications. Glycosylation consists of the addition of glycans (mono-, oligo- or polysaccharides) to a newly-formed peptide chain, regulating the structure and associated function of the final protein [26]. This is a dynamic, flexible, non-template driven and highly variable process, which can change dramatically upon alterations within the cellular milieu [26]. There are distinct glycosylation types in mammals that give rise to various glycoproteins categories such as *N*- linked, *O*-linked, GPI anchored or *O-*GlcNAc modified glycoproteins. The most abundant glycosylation class is the *N-*linked type since it has been reported that approximately 90% of eukaryotic glycoproteins carry *N-*glycans [27]. *N-*glycosylation is a multifaceted biosynthetic mechanism that starts in the endoplasmic reticulum and is completed in the Golgi, regulated by glycosidases and glycosyltransferases that determine the arrangement of the different glycosidic chains [28, 29]. The knowledge about *N*-glycans’ role in brain physiology is still limited; however, they are known to be involved in neuronal development and differentiation, synaptogenesis and myelinogenesis [30]. Nonetheless, the crucial role of *N*-glycans in the CNS is highlighted in the case of congenital disorders of glycosylation (CDGs), which result in multiple neuropathological symptoms that include epilepsy, seizures, stroke-like episodes and developmental delays [31]. This emphasises the need for an in-depth characterisation of the brain *N*-glycome, specifically in neurological conditions such as neuroinflammation, to understand how glycosylation might be playing a role in the pathogenesis of brain illnesses.

In the past decade, interest has grown in the role of glycosylation in neuroinflammation and on how the glycomic profile might be altered in this condition, suggesting a possible target for future therapies [32]. Only a few in vitro studies have been performed so far; however, all of them consistently describe a decrease in sialylation in inflammation models [33, 34]. This reduction in sialylation was also seen in an LPS-injected post-natal rat model [35]. Nonetheless, only perinatal infectious exposure was considered, and only one glycosylation trait was analysed (sialylation). This underlines the need and importance to evaluate neuroinflammation in the adult brain, covering the full *N-*glycome (as other glycosylation structures and features might play an essential role in this pathology).

Therefore, the aim of this study was to analyse the spatial modulation of the full *N-*glycomic profile in a rat model of neuroinflammation, using different glyco-analytical platforms such as liquid chromatography and mass spectrometry imaging. To establish a robust platform in which to characterise the *N-*glycome, we used a robust acute neuroinflammatory model induced by intrastriatal injection of LPS. Our results provide an insight into the glycosylation cues involved in inflammatory pathways (such as oligomannosylation, fucosylation and sialylation), and can help to identify targets to tackle it.

## Methods

**Fig. 1:**
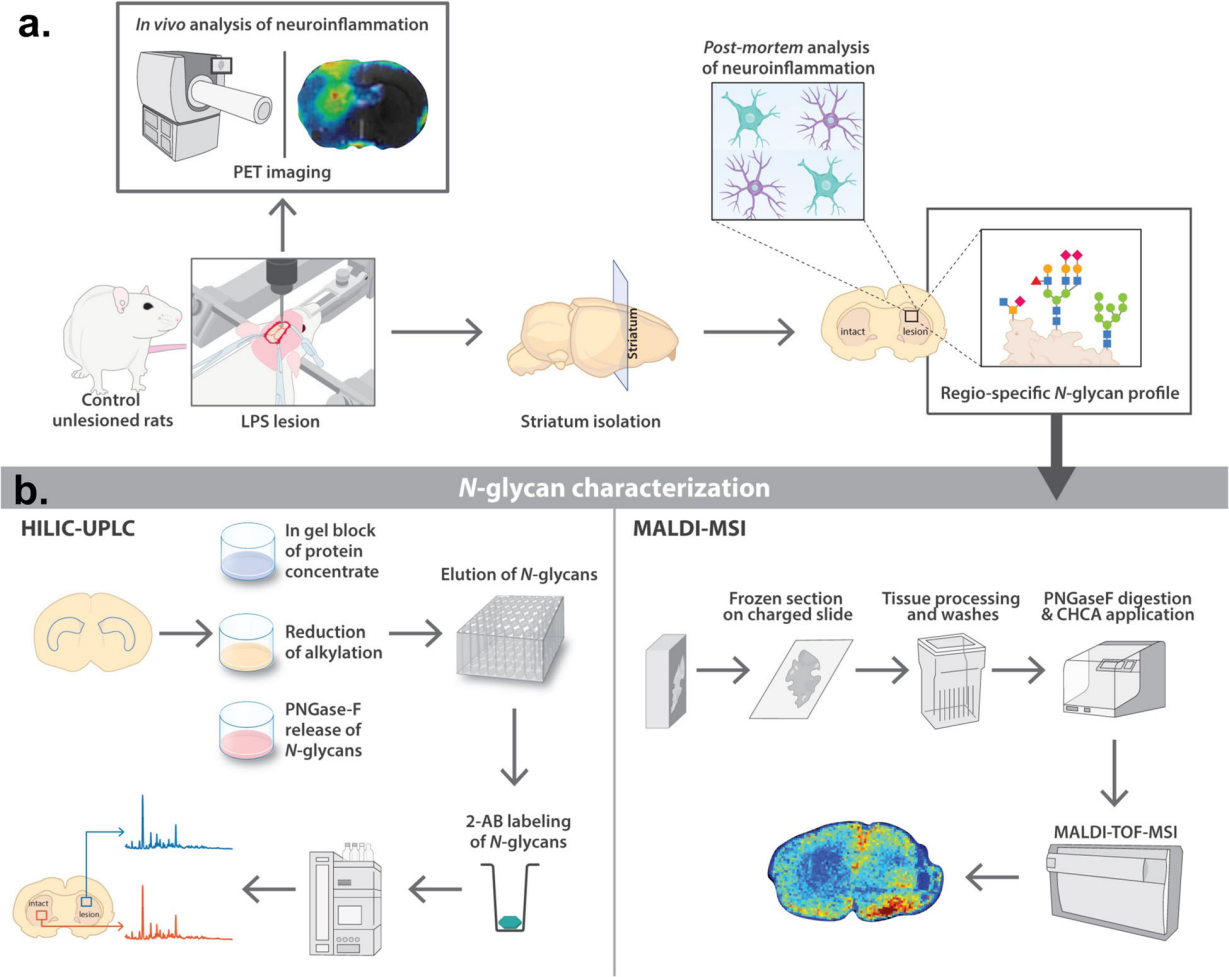
Schematic representation of the experimental design and procedures. **a** Establishment of a neuroinflammation model upon LPS injection in the rat striatum. The striatum was collected 7 days post-injection and further analysed for neuroinflammatory markers and *N*-glycomic profile. **b** Experimental plan of *N-*glycan characterisation. Regions of interest were either sectioned for MALDI-TOF-MSI or punched for *N*-glycan isolation and characterisation using HILIC-UPLC. The combination of both techniques allowed for the spatial and quantitative *N*-glycome profiling in the rat striatum

The study design is outlined in Fig. 1. Briefly, the striatal neuroinflammation model was established by unilateral injection of LPS into the rat striatum (10 μg), and inflammation was confirmed 1-week post-injection in vivo by positron emission tomography (PET) and then *post-mortem* through immunohistochemistry and gene expression of inflammatory markers (Fig. 1a). At this time point, brains were also collected to perform an in-depth quantitative analysis of the striatal *N-*glycome through hydrophilic interaction ultra-performance liquid chromatography (HILIC-UPLC), which was then confirmed spatially by matrix-assisted laser desorption ionisation mass spectrometry imaging (MALDI-MSI) (Fig. 1b). In all studies, the LPS-injected striatum was compared with the corresponding contralateral striatum to allow for a same-individual comparison.

### Animals

Adult male Sprague-Dawley rats (Janvier, France) of 8 weeks of age weighing 300–350 g were used in this study. Animals were housed in groups of two per cage, on a 12 h light/12 h dark cycle, at 21–23 °C and humidity 50%, with food and water available ad libitum throughout the whole experiment. This study was carried out following European Union (Directive 2010/63/EEC) and French (Act Rural Code R214/87-131; authorisation no. B92-032-02) regulations, and complied with Standards for Humane Care and Use of Laboratory Animals of the Office of Laboratory Animal Welfare (OLAW – n°A5826-01). All surgical procedures were reviewed and approved by the local ethics committee and registered with the French Research Ministry of Education and Research (committee no. 44, approval no. 12-100, APAFIS no. 389-20150327162135690v02). All efforts were made to minimise animal suffering and animal care was supervised by veterinarians and animal technicians skilled in rodent healthcare and housing.

### Surgeries

All surgical procedures were completed with animals under isoflurane anaesthesia (Iso-Vet; Coumon d'Auvergne, France) in O_2_, followed by a mixture of ketamine (75 mg/kg) and xylazine (5 mg/kg). Once anesthetised, animals were placed in the stereotactic frame and an intrastriatal injection of 2 μL of 5 μg/μL lipopolysaccharide (*n* = 30, LPS, L2880, Sigma, France) diluted in 1× Dulbecco’s phosphate buffer saline (DPBS, Gibco) or PBS (*n* = 12, 2 μL, supplementary figure S1a) was carried out. Injection coordinates were as follows [36]: Tooth bar: − 3.3 mm; anterio-posterior (basing on bregma): + 0.5 mm; lateral (basing on bregma): ± 3 mm; ventral (basing on dura): − 4.3 mm. The injection was achieved using an automatic pump (CMA-4004) with a speed of 0.5 μL/min, and a 34-gauge blunt-tipped needle connected by a polyethylene catheter to a 10 μL Hamilton syringe (Hamilton; Reno, USA). Once the infusion was completed, the needle was left in place for two minutes and then it was gently retracted.

### Positron emission tomography imaging

In vivo positron emission tomography (PET) imaging using [18F]DPA714 ([*N*,*N*-diethyl-2-(2-(4-(2-[18F]fluoroethoxy)phenyl)-5,7-dimethylpyrazolo[1,5-a]pyrimidin-3-yl)acetamide] was performed on LPS injected animals to image the 18 kD translocator protein (TSPO), a molecule located on the outer membrane of mitochondria, which is upregulated during inflammatory events [37]. The radiochemical purity of [18F]DPA714 was greater than 99% and specific radioactivity ranging from 100 to 165 GBq/μmol. [18F]DPA714 was formulated in physiological saline, at an injectable volume between 1.0 to 1.5 mL and the dose of 61 ± 13 MBq/mL (2.46 ± 1.65 nmol/mL; mean ± SD).

According to PET image acquisition and reconstruction procedures, rats (*n* = 9) were imaged 7 days post-injection (dpi) as previously described [38]. Briefly, animals were anaesthetised under isofluorane anaesthesia (Iso-Vet; Coumon d'Auvergne, France; 1.5–2.5% in 100% O_2_), and placed in a MicroPET® Focus 220 system (resolution: 1.5 × 1.5 × 1 mm; Siemens, France). A dynamic emission scan of 90 min was started simultaneously with the intravenous bolus injection of [18F]DPA714 (1 mL/min; Pump11 Elite, Harvard Apparatus Ltd.). The uptake of [18F]DPA714 was quantified by the non-displaceable binding potential (BPND) using a simplified quantification method (Ichise’s non-invasive plot, MRTM0 [39]), based on (pseudo-) reference regions (in this study: the contralateral striatum). The analysis was performed using PMOD® software.

### Tissue harvesting for immunohistochemistry

One week following LPS (or PBS, see supplementary figure S1) administration, and 1 day after PET imaging, animals were deeply anaesthetised by 4% isoflurane inhalation, followed by lethal injection of 180 mg/kg of sodium pentobarbital intraperitoneally. Depending on the analysis done, different procedures were followed for tissue processing. For immunohistochemistry analysis, animals were transcardially perfused with 280 mL of 4% paraformaldehyde (PFA) in 0.01 M phosphate-buffered saline (PBS). The brains were then collected, post-fixed for 24 h in 4% PFA at 4 °C and transferred to 30% sucrose in PBS afterwards. These were cut into coronal sections of 40 μm using a freezing-stage microtome (CM1900, Leica, Germany). Striatal sections were kept at − 20 °C in antifreeze solution (made of 30% sucrose, 30% ethylene glycol in PBS) until use.

### Immunohistochemistry

For free-floating immunohistochemistry (IHC), a previously established protocol was followed [40]. Briefly, sections were washed in PBS three times for 10 min and then incubated for 20 min with 0.3% H_2_O_2_ in PBS (at room temperature (RT)). After washing the sections again in PBS three times for 10 min, sections were blocked in 4.5% normal goat serum (NGS) in PBS with 0.2% Triton™ X-100 (PBST). Afterwards, sections were incubated with primary antibody in 3% NGS in PBST overnight at 4 °C. Primary antibodies used were GFAP (1:10.000; Z0334; Dako), Iba1 (1:3.000, 019_19741; Wako) and Vimentin (1:2.000; IF01; Calbiochem). Sections were washed in PBS and incubated in appropriate biotinylated secondary antibody (1:1.000; Vector Labs) diluted in 3% NGS in PBST for one hour at RT, followed by one hour incubation in Vectastain ABC kit (Vector Labs). Staining was revealed using the DAB peroxidase substrate kit with nickel (Vector Labs); sections were then washed in PBS, mounted onto glass slides and allowed to dry overnight. On the following day, sections were dehydrated by consecutive immersions in a gradient of ethanol baths (50%, 70%, 96%, 100%) and cleared twice in xylene before sealing with a coverslip and Eukitt mounting medium.

Stained sections were imaged using an ImageScanner III (GE Healthcare, USA) with Epson scan software (Epson, USA). Image analysis was done using FIJI (ImageJ software, NIH, USA). Briefly, images were transformed into a binary mode (8-bit format). The scale was set, areas of interest were manually outlined for each section (including a blank region) and light intensity of interest areas was measured. Afterwards, staining intensity was calculated as optical density (after subtracting the background) according to the equation:

$$ \left[ OD\right]=\mathit{\log}10\ \left(\frac{\max \mathrm{light}\ \mathrm{intensity}}{\mathrm{measured}\ \mathrm{light}\ \mathrm{intensity}\ \mathrm{sample}}\right)-\mathit{\log}10\ \left(\frac{\max \mathrm{light}\ \mathrm{intensity}}{\mathrm{measured}\ \mathrm{light}\ \mathrm{intensity}\ \mathrm{blank}}\right). $$

### Real-time quantitative PCR

For transcriptomic analysis, striata were dissected manually from the coronal 40 μm sections, using a surgical scalpel and a binocular microscope. mRNA was then extracted from these using E.Z.N.A.® FFPE RNA Kit (Omega Biotek, Georgia, USA) following the supplier’s heat extraction guidelines. mRNA levels were measured using Nanodrop (Thermo Fischer Scientific), and their quality was validated using Bioanalyzer (Agilent). According to the manufacturer’s instructions, mRNA was reverse-transcribed into cDNA using SuperScript™VILO™ cDNA Synthesis Kit (Vilo Life Technologies).

RT-qPCR was performed using iTaq™ Universal SYBR® Green Supermix (Bio-Rad) and primers (Eurofin Genomics) specific to different targets on 0.35–1 ng of cDNA, using 10 nM of primers (see Table 1). Reactions were run in triplicates in 384-well PCR plates, using a mix of cDNA and a housekeeping gene primer as an inter-plate control. Data were analysed using Bio-Rad CFX Maestro software (Bio-Rad). Cycle threshold (Ct) values were generated in regression mode. Results are shown as relative normalised expression.

**Table 1:** List of primers used for RT-qPCR

Gene/Primer	Forward sequence	Reverse sequence
Ppia/Cyclo (housekeeping)	ATGGCAAATGCTGGACCAAA	GCCTTCTTTCACCTTCCCAAA
Rplp0 (housekeeping)	CAGGCGTCCTCATTAGAG	ATCTGCTGCATCTGCTTGGAG
Hprt1 (housekeeping)	GGACCTCTCGAAGTGTTGGATAC	CCCTGAAGTGCTCATTATAGTCAA
GFAP	AATGACTATCGCCGCCAAC	CTCCTGGTAACTCGCCGACT
IBA1	CCAGCCTAAGACAACCAGCGTC	GCTGTATTTGGGATCATCGAGGAA
TNFα	AAATGGGCTCCCTCTCATCAGTTC	TCTGCTTGGTGGTTTGCTACGAC
TSPO/PBR	CAGTGTCCTTCACGGAGCAG	CGGGTACCCAGGATTGAGAC
Vimentin	GCAAAGCAGGAGTCAAACGA	AATTCTCTTCCATTTCACGCATCT

### Tissue extraction and homogenisation for *N*-glycome analysis

For *N-*glycome analysis through liquid chromatography-based methods, snap-frozen tissue was used. Intact brains were collected as previously mentioned and mounted onto a cryostat chuck using optimal cutting temperature compound (OCT; Sigma, Ireland) and fully sectioned into 200 μm slices in a Microm HM 505 E cryostat (GMI; USA). These were collected on SuperFrost™ Plus Adhesion charged slides (Fischer Scientific, Ireland) where it was easier to identify the sections containing striatum using a rat brain atlas. To isolate and extract these regions, micron biopsy punches of 2 mm and 0.5 mm in diameter (Harvard apparatus; USA) were used. This procedure was carried out entirely inside the cryostat, at − 20 °C. Striatal tissue punches were collected, weighed and stored at − 80 °C until being processed further.

The snap-frozen striatal tissue was homogenised in RIPA buffer (Sigma, Ireland) and cOmplete Protease Inhibitor Cocktail (Roche, Ireland, 1:25) using the QIAGEN TissueLyser LT (QIAGEN, UK), during 8 min at 40 Hz (at 4 °C). The homogenates were centrifuged for 20 min at 16,000 *g* (4 °C) and the supernatants collected and kept at − 80 °C until further use.

### *N-*glycan analysis by liquid chromatography

#### Materials and reagents

For *N-*glycan release and analysis by liquid chromatography, the following materials and reagents were used. AcroPrep Advance 96-filter plates and 10 kDa MWCO microcentrifuge filtration tubes were acquired from Pall Life sciences (USA). Polypropylene 2 mL deep 96-well blocks were purchased from Fisher Scientific (Ireland) and 0.45 μm Millex-LH filters from Millipore (Ireland). Plate seals were obtained from Cruinn (Ireland) and silicone sealing mats from Phenomenex (Germany). Protogel was purchased from National Diagnostics (UK) and ammonium hydroxide solution was acquired from Fluka (Ireland). Formic acid and ammonium persulfate (APS) were obtained from VWR chemicals (Ireland). Ultrapure water was filtered through an arium® ProUV system (Sartorius, Germany). HPLC grade methanol, ethanol, acetonitrile, xylene and water were acquired from Fisher Scientific (Ireland). PNGase F was purchased from New England Biolabs. All other reagents were purchased from Sigma (including *N*,*N*,*N*′,*N*′-tetramethylethane-1,2-diamine (TEMED), iodoacetamide (IAA), dithiothreitol (DTT) and 2-aminobenzamide (2-AB)) unless otherwise specified.

#### Release of *N*-glycans

The isolated glycoproteins from brain tissue were dried in a vacuum centrifuge overnight (Savant™ SPD131DDA SpeedVac™ Concentrator, Fisher, Ireland) and *N-*glycans released from dried tissue as described previously [41]. Briefly, the glycoproteins were dissolved and then immobilised in acrylamide gels made of protogel and sodium dodecyl sulfate (SDS). The gels were cut and washed, followed by reduction and alkylation of the proteins with DTT and IAA, respectively, in 96-well plates. This allowed for the disulphide bonds from the protein to be disrupted, exposing *N-*glycan residues. *N*-glycans were cleaved using PNGase F (1239U/mL, New England BioLabs, Inc., UK) [42]. These were then reduced in formic acid.

#### 2-AB labelling of *N*-glycans

Released *N*-glycans were fluorescently labelled with 2-aminobenzamide (2-AB) by reductive amination [43, 44]. Briefly, the 2-AB labelling solution containing sodium cyanoborohydride was added to the glycans and incubated at 65 °C for 2 h. The excess 2-AB solution was removed by absorption on Whatman 3MM paper (Merck, Ireland) in acetonitrile washes, being the clean labelled glycans eluted in water [45].

#### Hydrophilic interaction ultra-performance liquid chromatography

Labelled *N*-glycans were analysed by hydrophilic interaction ultra-performance liquid chromatography (HILIC-UPLC). This was carried out using a UPLC Glycan BEH Amide Column, 130 A, 2.1 × 150 mm, 1.7 μm particles (Waters, USA) on an H Class Acquity UPLC system (Waters, USA) assembled with a Waters Acquity fluorescence detector and a Waters temperature control module. This was used to keep the column temperature at 40 °C and the sample temperature at 5 °C. Solvents A (50 mM ammonium formate, pH 4.4) and B (acetonitrile) were used in a method that had a duration of 30 min. The method consisted of a linear gradient of 30% to 47% of solvent A for 24 min at 0.561 mL/min flow rate, increasing to 70% at minute 25 and returning to 30% at minute 27 until the end of the run. Samples were suspended and injected in 70% acetonitrile. Once in the system, these were excited at 330 nm and fluorescence recorded at 420 nm. A 2-AB labelled dextran calibration ladder with glucose oligomers (Waters, USA) was included as an internal standard at the beginning of each sample set, as previously described [44].

### Tissue processing for MALDI-MSI

For *N-*glycome analysis through mass spectrometry imaging, frozen 10 μm serial sections of brain tissue on SuperFrost™ Plus Adhesion charged slides (Fischer Scientific, Ireland) were used. However, these had to be transformed before being used for imaging. Briefly, sections were thawed for 15 min and dehydrated in serial dilutions of ethanol (70%, 90%, 100%, 100%) for 2 min each. Afterwards, sections were incubated at 60 °C for 50 min, followed by delipidation in Carnoy solution twice (60% ethanol, 30% chloroform, 10% glacial acetic acid), for 3 min, and 2 min wash in running tap water. Finally, these were incubated for 30 min with 10% formalin solution, neutral buffered (Sigma, Ireland) at room temperature, followed by two washes in tap water. The samples were then air-dried and kept at room temperature in a desiccator until further analysis.

### *N-*glycan analysis by MALDI-MSI

#### Antigen retrieval

The previously transformed slides were subjected to antigen retrieval using citraconic anhydride buffer (ThermoScientific™, USA), prepared by mixing 25 μL of citraconic anhydride in 50 mL of HPLC grade water, adjusted to pH 3 with 12 M HCl. Slides were incubated in this buffer for 30 min in a vegetable steamer (around 95 °C) and then washed (after cooling) in serial dilutions of the buffer by replacing half of the buffer with HPLC grade water, three times, eventually replacing it entirely with water. The slides were desiccated and scanned before applying PNGase F.

#### Application of PNGase F and CHCA matrix

After antigen retrieval, slides were coated with 0.25 mL aqueous solution of recombinant PNGaseF (Bulldog Bio, USA) at 0.1 μg/μL, spraying at 25 μL/min in 15 passes at 45 °C, using an HTX TM-Sprayer (HTX Imaging, USA) as previously described [46]. This was followed by an incubation of two hours at 37 °C in a humidified chamber and placed in the desiccator until sprayed with matrix (ideally on the same day). α-Cyano-4-hydroxycinnamic acid (CHCA) matrix was prepared fresh (7 mg/mL in 50% acetonitrile 0.1% TFA) and applied on the sections at 100 μL/ min in 10 passes at 80 °C using an HTX TM-Sprayer (HTX Imaging, USA). Coated slides were stored in a desiccator until analysed.

#### MALDI-MSI analysis set up

Released *N-*glycan ions were detected using a MALDI timsTOF fleX trapped ion mobility separated QTOF mass spectrometer (Bruker Daltonics, Germany) operating in a positive mode as described by McDowdell et al. [47]. This had a SmartBeam 3D laser operating at 10 kHz and a laser spot size of 20 μm. Signal was collected at a raster width of 40 μm between spots. A total of 300 laser shots were collected to form each pixel. Following acquisition, data was processed, and images of expressed glycans were generated using FlexImaging 5.0 and SCiLS Lab 2017b software (Bruker Daltonics, Germany), where ions in the range of 500–4000 m/z were analysed. Observed mass/charge ratios (*N-*glycans) were searched against glycan databases using GlycoWorkbench. Represented glycan structures were also generated in GlycoWorkbench, as they were determined by a combination of their measured accurate m/z, CID fragmentation patterns and previous structural characterisation carried out by UPLC (as described previously).

### Glycan nomenclature

*N*-glycans share a common pentasaccharide with two core *N-*Acetylglucosamine (GlcNAc) and three mannose residues. F indicates a fucose residue, so if it is placed at the start of the abbreviation, it refers to core α(1,6)-fucose linked to the inner GlcNAc, whereas if it appears anywhere else, it indicates an outer arm α(1,3) or α(1,4)-fucose linked to antenna or galactose. Ax indicates the number (x) of antenna (GlcNAc) on the mannose residues belonging to the *N-*glycan core. Gx refers to the number (x) of β(1,4)-linked galactose on the antenna and Galx to the number (x) of α(1,3/4/6)-linked galactose on β(1,4)-linked galactose. Sx relates to the number (x) of α(2,3/6/8)-linked neuraminic acids (sialic acids) connected to galactose, while Sgx stands for the number (x) of glycolylneuraminic acids linked to galactose. Mx refers to the number (x) of mannose residues on the core GlcNAc (terminology used in oligomannose glycans). Lacs concerns the number (x) of poly-*N-*Acetyllactosamine repeats consisting of GlcNAc β(1,4)-linked to galactose.

### Statistical analysis

Data were processed using GraphPad Prism8 software. Immuno-histochemistry data, qPCR data and PET imaging data are expressed as mean ± SEM. These were analysed using paired Student’s *t* test to compare LPS-injected and non-injected (NI)/contralateral striata in the same animal. *N-*glycome data was also analysed using paired Student’s *t* test, after Log-transforming each glycan peak’s abundance to obtain a normal distribution. The statistical significant difference was set at *p < 0.05, **p < 0.01, ***p < 0.001, ****p < 0.0001, and significance is expressed between glycosylation levels in LPS-injected striatum vs NI striatum. *n* indicates the number of rats.

## Results

### LPS-injected striatum displays neuroinflammatory hallmarks in vivo and post-mortem

**Fig. 2:**
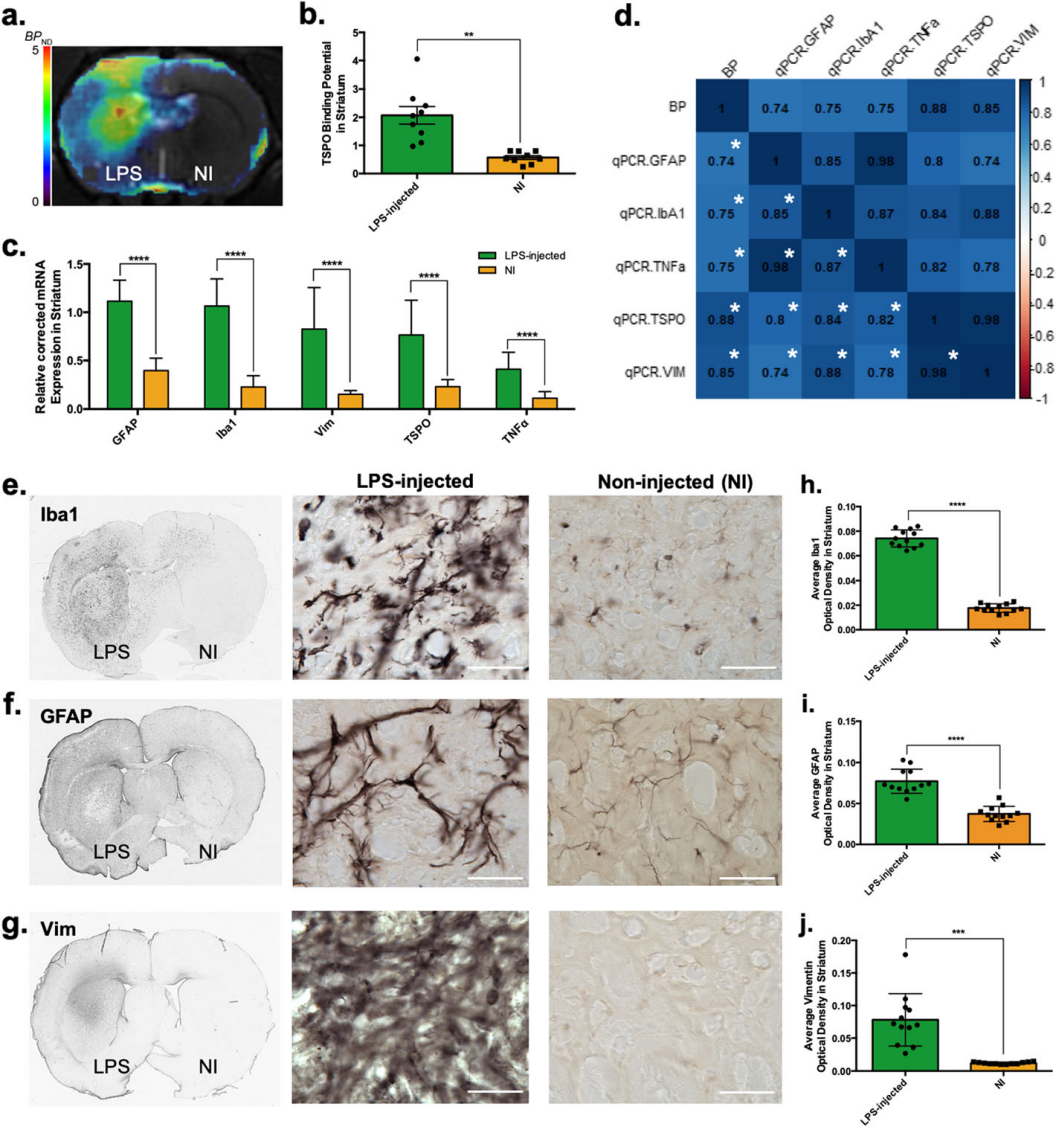
Pathological validation of the model of striatal neuroinflammation where LPS was injected into the striatum, in comparison to the contralateral (non-injected (NI)) striatum at seven days post-injection (dpi). **a** In vivo analysis of translocator protein (TSPO) expression as a marker of neuroinflammation in the striatum post-LPS injection. Average quantified TSPO-PET image in non-displaceable binding potential (BP_ND_) in a coronal section. **b** Quantification of TSPO-PET imaging in the striatum. Results are expressed as means ± the standard error of the mean (SEM). *n* = 9; paired Student *t* test and statistically significant difference was set at **p < 0.01. **c** Striatal mRNA expression of different genes related to the inflammatory response–Glial fibrillary protein (GFAP), Iba1, vimentin (Vim), TSPO and tumour necrosis factor α (TNFα). Results are expressed as means ± SEM. *n* = 11–12; paired Student *t* test and statistically significant difference was set at ****p < 0.0001. **d** Spearman correlation between in vivo individual PET BPnd data and *post-mortem* expression data. Spearman correlation coefficients are marked in the corresponding case; blue signifies a positive correlation. Statistical significance was set at *p < 0.01. **e**, **f**, **g** Histological evaluation of the expression of Iba1, GFAP and Vim (respectively) in LPS-injected vs non-injected striata at seven dpi. Scale bar = 50 μm. **h**, **i**, **j** Striatal optical density of Iba+, GFAP+ or Vim+ (respectively) in the LPS-injected and NI striata. Results are expressed as means ± SEM. *n* = 10–12; paired Student *t* test and statistically significant difference was set at ***p < 0.001, and ****p < 0.0001

To establish a robust and reliable platform in which to characterise the *N-*glycome, a rat acute model of striatal neuroinflammation was optimised (Fig. 2). In vivo PET imaging of the 18 kD TSPO showed a statistically significant increased BP_ND_ in the LPS-injected striatum compared to the non-injected (NI) contralateral side (Fig. 2a, b, *n* = 9, Paired Student’s *t* test, *p* < 0.0011). This was correlated with the TSPO mRNA expression, where a significant increase was also detected by RT-qPCR in the LPS-injected side (Fig. 2c, *n* = 11, *R* = 0.87, Paired Student’s *t* test, *p* < 0.0004).

In vivo data was correlated with *post-mortem* data through Spearman correlation analysis, showing positive associations between all different inflammation-related markers (Fig. 2d), between 0.74 (GFAP mRNA expression vs TSPO BP_ND_) and 0.85 (Vim mRNA expression vs TSPO BP_ND_).

*Post-mortem* analysis of inflammatory markers [glial fibrillary acid protein (GFAP), ionised calcium-binding adapter molecule 1 (Iba-1) and vimentin (Vim)], both by mRNA expression through RT-qPCR (Fig. 2c) and by IHC analysis (Fig. 2e–j) confirmed LPS induced localised neuroinflammatory reaction. The density of activated microglia, as detected by IHC of the protein Iba1, was significantly increased in the LPS-injected striatum as compared to NI control striatum (+ 322 ± 25%, *n* = 12, Paired Student’s *t* test, *p* < 0.0001) (Fig. 2e, h). In parallel, increased presence of astrocytes (GFAP and Vim) was observed in the LPS-injected striatum compared to the NI striatum [GFAP IHC (+ 107 ± 11%, *n* = 12, Paired Student’s *t* test, *p* < 0.0001) (Fig. 2f, i); and vim IHC (+ 544 ± 238%, *n* = 12, Paired Student’s *t* test, *p* < 0.0001) (Fig. 2g, j)]. These results were coherent with the mRNA expression data (Fig. 2c) for each of these markers, where a dramatic increase of Iba1(+ 371%, *n* = 11, Paired Student’s *t* test, *p* < 0.0001), of GFAP (+ 181%, *n* = 10, Paired Student’s *t* test, *p* < 0.0001) and of vimentin (+ 454%, *n* = 10, Paired Student’s *t* test, *p* = 0.0002) was seen. Additionally, mRNA expression of the pro-inflammatory cytokine Tumour necrosis factor α (TNFα) was also significantly increased in the ipsilateral striatum (+ 277%, *n* = 10, Paired Student’s *t* test, *p* = 0.0003).

To confirm that LPS was responsible for the neuroinflammatory reaction, a parallel study was done on a subgroup of PBS-injected animals (Supplementary figure S1a). Immunohistological analysis in the PBS-injected striatum of the inflammatory markers (Iba1, GFAP, Vim) did not show any significant increase in the density of any of these markers as compared to the NI striatum (Figure S1c-h) [Iba1: *n* = 6, Paired Student’s *t* test, non-significant (NS); GFAP: *n* = 6, Paired Student’s *t* test, NS; Vim, *n* = 5, Paired Student’s *t* test, NS]. mRNA expression of Iba1 and GFAP was also not altered in the PBS-injected striatum compared to contralateral NI side (Figure S1b; Iba1: NS, *n* = 7, Paired Student’s *t* test; GFAP: NS, *n* = 6, Paired Student’s *t* test). The expression of TNFα mRNA was increased in the PBS-injected striatum compared to contralateral NI side (Figure S1b; *n* = 6, Paired Student’s *t* test, *p* = 0.0071). However, the expression of TNFα after PBS injection was lower than the LPS-injected striatum (TNFα mRNA expression was 25% higher in the LPS-injected striatum in comparison to the PBS-injected striatum). Altogether, these data confirmed that LPS induces a significant neuroinflammatory reaction, providing a robust and reproducible model.

### *N-*glycome profile in the LPS-injected striatum is significantly different from that in the contralateral (non-injected) hemisphere

**Fig. 3:**
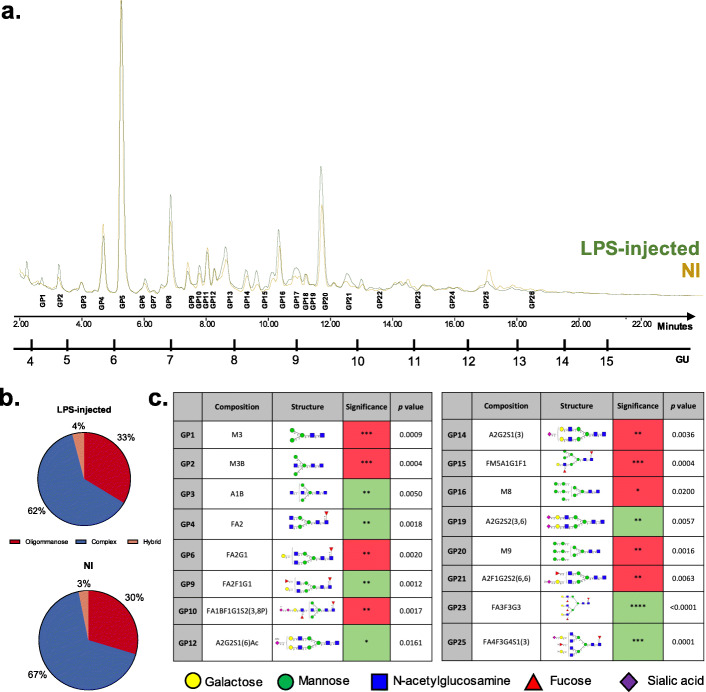
*N*-glycome changes between LPS-injected and NI striata using HILIC-UPLC. **a** HILIC-UPLC chromatograms for *N-*glycans isolated from rat striata (LPS-injected vs NI) during a 30-min run, separated into 26 main chromatographic glycan peaks (GP) following the characterisation performed by Samal et al. [41]. Detailed composition of each of these peaks is described in Supplementary table S1. **b** Relative abundances represented as percentages of total *N*-glycans divided into the three main biosynthetic classes: oligomannose, complex and hybrid. **c** Summary table of the GP that are statistically significantly different between LPS-injected and NI striatum at seven dpi. Red indicates significantly increased peak area (abundance) in the LPS-injected striatum, whereas green represents significantly decreased peak area in the LPS-injected striatum, compared to NI striatum. The abundance of these was Log transformed for statistical analysis. *n* = 5; paired Student’s *t* test was used to compare groups in each GP. All other GPs (not mentioned) did not show any significant difference in abundance between LPS-injected and NI striata

To quantitatively analyse the changes in *N-*glycome seen in the LPS-injected striatum, hydrophilic interaction ultra-performance liquid chromatography (HILIC-UPLC) was used as previously described [41]. This provided the chromatographic profile of *N-*glycans isolated from the glycoproteins present in the LPS-injected and NI striata, after releasing them using PNGase F and labelling them with 2-AB (Fig. 3). Chromatograms of 26 glycan peaks (GP) were observed (Fig. 3a, Supplementary table S1). When pooled into the main biosynthetic classes, there is a decrease in complex structures and an increase in oligomannose, as would be predictable from the individual GPs changes (Fig. 3b).

From the 26 GPs identified, 16 of them were significantly different between LPS-injected and NI striata (Fig. 3c). From these, nine were significantly increased following LPS injection, mainly oligomannosylated structures (Fig. 3c, M3: *n* = 5, Paired Student’s *t* test, *p* < 0.0009; M3B: *n* = 5, Paired Student’s *t* test, *p* < 0.0004; M8: *n* = 5, Paired Student’s *t* test, *p* < 0.02; M9: *n* = 5, Paired Student’s *t* test, *p* < 0.0016). The GPs significantly decreased were mainly sialylated (Fig. 3c, A2G2S1Ac: *n* = 5, Paired Student’s *t* test, *p* < 0.0161; A2G2S2: *n* = 5, Paired Student’s *t* test, *p* < 0.0057; FA4F3G4S1: *n* = 5, Paired Student’s *t* test, *p* < 0.0001).

**Fig. 4:**
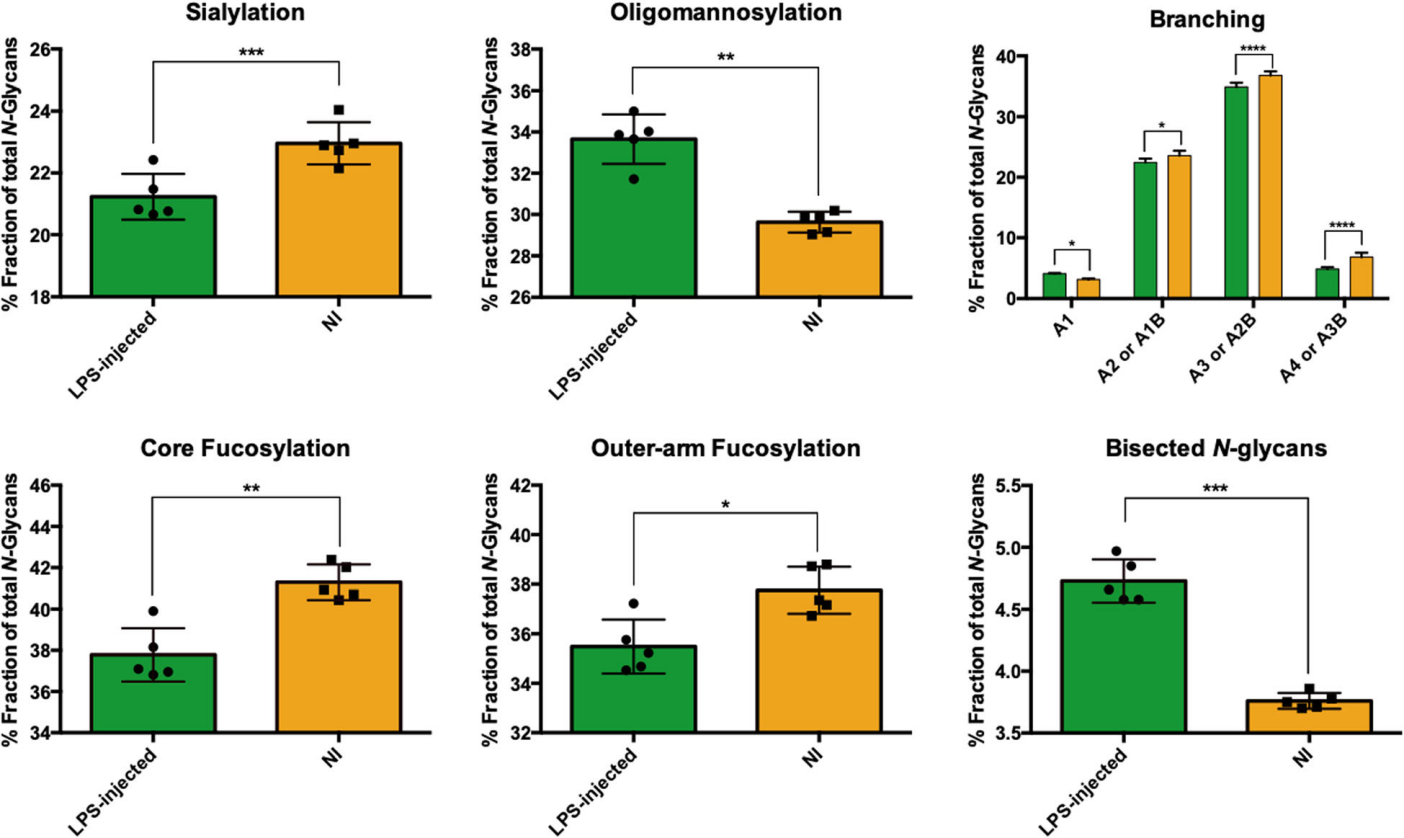
Changes in the *N*-glycosylation traits between LPS-injected striatum and contralateral (non-injected (NI)) striatum at seven days post-injection. The common glycosylation features amongst the main structures in each glycan peak were grouped in the main glycosylation traits, according to Supplementary table S3. Only the most abundant glycan in each GP was considered. Data presented as the mean ± SD, *n* = 5. Paired Student’s *t* test was used and statistically significant difference was set at *p < 0.05; **p < 0.01; ***p < 0.001; ****p < 0.0001

Looking in greater depth into the main glycosylation features, GPs were grouped into traits such as sialylation, core fucosylation, outer-arm fucosylation, branching degree and bisected *N-*glycans (Fig. 4) following the calculation rationale described in Supplementary table S3. It was seen that in all of these there were significant differences: a drastic decrease in sialylation (*n* = 5, Paired Student’s *t* test, *p* < 0.0005), in core fucosylation (*n* = 5, Paired Student’s *t* test, *p* < 0.0044), in outer-arm fucosylation (*n* = 5, Paired Student’s *t* test, *p* < 0.0131) and in structures with 2, 3 or 4 antennae (or bisecting monoantennary, bisecting diantennary or bisecting triantennary, respectively) (A2/A1B: *n* = 5, Paired Student’s *t* test, *p* < 0.0405; A3/A2B: *n* = 5, Paired Student’s *t* test, *p* < 0.0001; A4/A3B: *n* = 5, Paired Student’s *t* test, *p* < 0.0001) were seen after LPS injection. In contrast, there was a significant increase in oligomannose structures (*n* = 5, Paired Student’s *t* test, *p* < 0.0021) and in bisected *N-*glycans (*n* = 5, Paired Student’s *t* test, *p* < 0.0002).

### Sialylation and fucosylation are the main traits dysregulated upon neuroinflammation

**Fig. 5:**
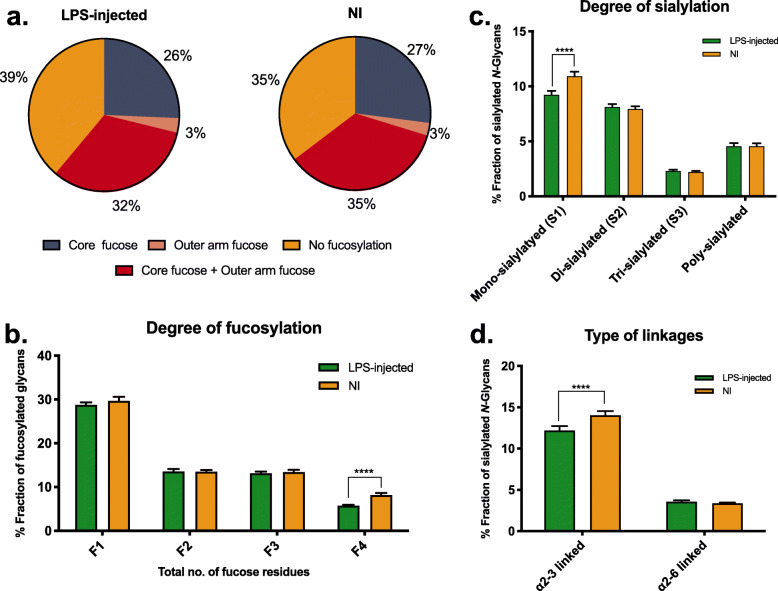
Changes in the percentage fraction of fucosylated and sialylated *N-*glycans between LPS-injected striatum and contralateral (non-injected (NI)) striatum at seven days post-injection. **a** Proportion of total *N*-glycans decorated with either core or outer arm fucose (or both), considering the main glycan structure in each GP. **b** Percentage fraction of fucosylated *N*-glycans according to the degree of fucosylation (*n* = 1, 2, 3, 4), which includes both sialylated and unsialylated fucosylated glycans. Data presented as the mean ± SD, *n* = 5. Paired Student’s *t* test was used, and statistically significant difference was set at ****p < 0.0001. **c** Percentage fraction of sialylated *N*-glycans according to the degree of sialylation, considering the main glycan structure in each GP. **d** Percentage fraction of sialylated *N*-glycans according to the linkage of the sialic acid to galactose residue indicates the significantly high abundance of α(2-3)-linkage to galactose compared to that of α(2-6)-linkage. Data presented as the mean ± SD, *n* = 5. Paired Student’s *t* test was used, and statistical significant difference was set at ****p < 0.0001

General discrepancies in sialylation and fucosylation have been reported in CNS-impairments and neuroinflammatory scenarios [35, 48]. Therefore, we looked further into these two features in the context of *N-*glycosylation (Fig. 5). Regarding fucosylation, a decrease was seen in both outer arm and core fucosylation, as mentioned above (Fig. 5a). However, the main structures affected were the heavily fucosylated ones (with four fucose residues—F4; *n* = 5, Paired Student’s *t* test, *p* < 0.0001) (Fig. 5b). Interestingly, in the case of sialylation, the decrease seen in the LPS-injected group seems to be due to a lower abundance in mono-sialylated *N-*glycans (*n* = 5, Paired Student’s *t* test, *p* < 0.0001) (Fig. 5c). Additionally, the primary type of linkages affected is the α2,3 (*n* = 5, Paired Student’s *t* test, *p* < 0.0001), whereas sialic acid bound through α2,6 linkages seems to remain unaffected (Fig. 5d).

### Spatial distribution of *N-*glycome in LPS injected striatum is region-dependent

To elucidate the spatial distribution of the brain *N-*glycophenotype upon LPS injection, MALDI-MSI of *N-*glycans was used as described by Powers et al. [49]. In this case, fixed-frozen tissue sections were used, and, after antigen retrieval, sections were sprayed with PNGaseF, followed by α-Cyano-4-hydroxycinnamic acid (CHCA matrix) to facilitate ion detection. The glycan composition analysis was performed by identifying the mass to charge ratio of each peak and comparing it to a database in GlycoWorkBench with all possible mammalian *N-*glycan compositions. These were further compared with the databases from the Consortium for Functional Glycomics (www.functionalglycomics.org) for comparison with previous studies and discard any biologically irrelevant matches. This promoted the validation of the data previously detected by HILIC-UPLC and further refinement and in-depth elucidation of the rodent striatum *N*-glycome. An intra-animal comparison was performed to follow the analysis done previously.

**Fig. 6:**
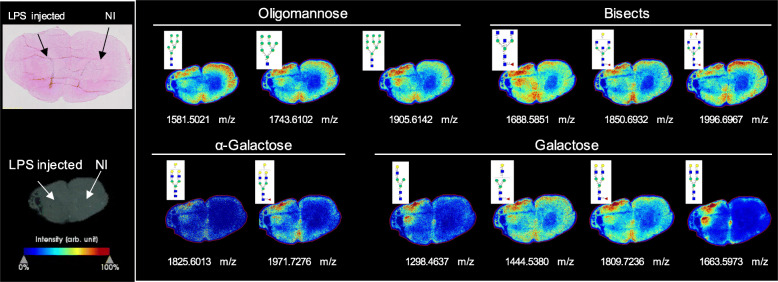
*N*-glycan imaging of the brain of LPS-injected rats. Main *N*-glycan structures that are visualised in the lesion core belong to specific groups: oligomannose, bisected structures and galactosylated glycans, mainly with α-galactose. Each image is accompanied by the putative structures determined by combinations of accurate m/z, CID fragmentation patterns and glycan database structure

Overall, a total of 52 *N-*glycans were detected by MALDI-MSI (panels of all detected *N-*glycans through MALDI-MSI are described in Supplementary Figure S2, Supplementary Figure S4, Supplementary Figure S5 and Supplementary table S2), providing a high-resolution spatial distribution of *N*-glycans in the rodent striatum. There are some dramatic changes seen in the injection site, directly correlated with the inflammatory reaction (Fig. 6). These changes are mainly characterised by the marked increase in the expression of oligomannose, bisected and α-galactosylated structures in the lesion core (i.e. in the striatum, and across the needle tract in the cortex).

On the other hand, some structures seem to be depleted upon neuroinflammation, not only in the striatum but in the whole hemisphere (including the cortex, where minor signs of inflammation are also seen—Fig. 2e–g). Overall, these include the fucosylated and sialylated structures, which seem to be down-regulated in the LPS-injected side (Supplementary Figure S4), following the UPLC data trends. Regarding the 16 glycan structures whose abundance was seen to be significantly different between LPS-injected and NI striata, 10 of them were detected by MALDI-MSI (Supplementary Figure S3). Five out of the six non-detected structures are sialylated; however, due to the low abundance and instability of sialic acids, which can be readily lost in MSI steps [50, 51], both in the ion source and during the flight to the detectors, this would be expected. The detected GP’s spectra were plotted according to their intensity in each striata, and it was seen that the trends in intensity (LPS-injected vs. NI striata) are similar to the significant changes indicated by HILIC-UPLC (Supplementary Figure S3). This intra-animal comparison confirms the highly flexible and dynamic nature of glycosylation depending on the cellular milieu. In the same animal (different hemispheres), there is a significant difference in the *N-*glycomic profile, depending only upon the administration of LPS. Furthermore, the injection site’s distance was also seen to impact the *N-*glycomic profile as it can be seen in Supplementary figure S6. The further away we move from the injection site, the more similar the *N-*glycomic profile is to the contralateral side, emphasising the influence of LPS-induced neuroinflammation on the modulation of the *N*-glycome.

## Discussion

*N-*glycosylation is a type of post-translational modification whose dysregulation in the brain dictates many conditions ultimately related to neuronal dysfunction [52, 53]. However, the *N-*glycophenotype of distinct neurological scenarios is still unknown, representing a gap in the in-depth knowledge of such diseases. To date, this is the first study to explore the complete modulation of the *N-*glycome upon neuroinflammation in the rodent brain. It was hypothesised that the spatial distribution of *N-*glycans in the striatum of a rodent model of neuroinflammation gives an insight into the glycosylation cues involved in the inflammatory pathways and can help to identify potential targets to tackle it. To test this hypothesis, firstly an in vivo acute model of neuroinflammation was optimised to ensure reproducibility of the lesion and consistency in the results. This was followed by quantitative analysis of the changes in the expression of different *N-*glycan structures between LPS-injected and non-injected striata, which was finally validated spatially by assessing the distribution of the *N-*glycome in the brain of this preclinical model, using different glyco-analytical platforms such as HILIC-UPLC and MALDI-MSI. This follows a previous study by our group where the detailed and specific *N-*glycosylation profiles of the rat striatum and substantia nigra were comprehensively characterised [41].

A multifaceted approach combining in vivo and *post-mortem* tools on the same animals was undertaken to measure inflammatory-related markers in the ipsilateral striatum to characterise the neuroinflammation model*.* Significantly increased TSPO binding in vivo using [18F]DPA714 PET imaging was seen in the LPS-injected striatum, which most likely related to an increase in pro-inflammatory TSPO-expressing activated microglia and astrocytes [38, 54, 55]. *Post-mortem* analysis of the tissues through immunohistochemistry confirmed the significantly higher density of microglia and reactive astrocytes and confirmed their morphological changes typical of a reactive phenotype. This is in coherence with previous studies demonstrating that LPS-induced pro-inflammatory microglia can induce astrocytes activation [56]. In our study, we were able to see that astrocytes are significantly more expressed in an acute neuroinflammation model, even if at a lower abundance than that of microglia. Additionally, there is a strong association between the expression of TSPO and GFAP/Vimentin (astrocytic markers), highlighting the correlation between them.

Another crucial hallmark of neuroinflammation, in addition to increased microglial density, is the secretion of pro-inflammatory cytokines and other neurotoxic factors [57], which have a deleterious effect on the neuronal circuitry [58]. This neuronal damage feeds further into a positive feedback loop, which promotes further inflammation. Our results show a significant increase in the production of TNFα post-LPS-injection that can be mainly secreted by microglia and infiltrating macrophages, which express CD68 [59]. This supports the previous reports, such as the LPS model established by Ory and colleagues, which was described to increase CD68^+^ cells significantly in the LPS-injected striatum [60]. Beier et al. also showed an increase in activated microglia and CD68^+^ cells in the brain after systemic and repeated injection of LPS [61]. Additionally, the model implemented by Herrera et al. showed an increase in integrin alpha M (ITGAM)-positive cells with macrophage morphology in the core of the lesion [62], also corroborating what we described and emphasising that the PET signal seen is most likely due to microglia/macrophages contribution. These results confirm the establishment of an optimised robust acute neuroinflammatory model that with a moderate dose of LPS (10 μg) can induce high expression of reactive astrocytes and microglia and other inflammatory markers such as TNFα and TSPO.

The effect of glycosylation in inflammatory cascades and how this is modulated during the different neuroinflammation phases has been of increased research interest in recent years. A clear example comes from the pro-inflammatory activity of TNFα, which is predominantly mediated by TNF receptor 1 (TNFR1), whose *N-*glycosylation was reported to be altered in microglia [63]. This facilitates the interaction and increases binding-affinity of TNFα to this receptor, promoting further an autocrine loop in microglia and increasing the inflammatory cascades.

Having established a reproducible neuroinflammation model, it was possible to use it to investigate the modulation of tissue *N*-glycosylation in this scenario. Even though some in vitro studies were conducted in different models to investigate glycan changes, most of these studies explore only specific glycosylation traits (e.g. sialylation), or are not focused on a type of glycosylation (such as *N-*glycans or *O*-glycans), but rather on the overall glycomic signature. To date, only one in vivo study was carried out to explore a single glycosylation trait in a model of neuroinflammation [35], leaving a gap in this knowledge. Furthermore, studying the *N-*glycome of the overall tissue instead of specific glycoproteins offers the advantage of promoting therapeutic approaches that can attenuate these global dysregulations, rather than targeting individual molecules, which is exponentially more complex.

Looking at the modulation of the overall *N-*glycosylation traits upon LPS-injection, the decrease in sialylation comes as an interesting and expected result. Sialic acids are crucial players in CNS homeostasis, axonal guidance and neuronal growth [64, 65], mainly for their polarisation (negative charge). They modulate the voltage-gated ion channels (VGICs) activity in an isoform-specific manner according to the cell type, environment, and developmental stage [66]. This residue’s importance is probably why most glyco-targeted studies in the brain are focused on sialylation. For example, it was seen that mice deficient in sialyltransferases St3gal2 and St3gal3 (mainly involved in the assembly of gangliosides) presented motor impairments and cognitive deficits, besides increased dysmyelination [67], indicating the crucial role of sialylation. As LPS impacts microglia, which modulates neuronal function and synaptic maintenance, a decrease in sialylation could be involved in the dysregulation of signal transmission. Sumida et al. reported that LPS-activated microglia display upregulated secretion of a sialidase (NEU1) that cleaves polysialic acid (PSA) in neural cells [34], which comes in line with the decrease in sialylation that we found in the present study. This suggests that the increase seen in the abundance and size of microglia and its reactivity could be involved with decreased sialylation through the elevated secretion of sialidases (enzymes responsible for cleaving sialic acid residues) [68] or decreased secretion of sialyltransferases. Similar findings were seen in vivo, in a post-natal neuroinflammatory model (LPS injected), where a significant upregulation in sialidases was described, leading to decreased sialylation [35].

In an in vitro model where reactive astrocytes and neurons were used, it was reported that α2,6-sialic acid is absent in the healthy neuronal culture, but present in neurons in the injured environment. This indicates that changes in sialylation might be a consequence of the injury and that sialic acid could be contributing to the pathology [69]. The low abundance of α2,6-sialic acid in the striatum revealed no significant differences in our study, which might suggest that in a complex in vivo environment, these do not play such a crucial role. On the other hand, it has been reported that *N-*glycans in the adult brain display predominantly α2,3-linked sialic acids [52, 70, 71], which is consistent with what we have described. The significant decrease in this structure upon LPS-injection might be related to dysmyelination and neuronal impairment, as α2,3-linked sialic acids have been reported to be essential players in these physiological functions [52]; however, further studies would be required to assess if our model presents dysmyelination.

The presence of structures containing polysialic acids (PSA) is also of interest as these are almost exclusive of neural cell adhesion molecule (NCAM), linked through α2,8 linkages, appearing also associated to voltage-dependent sodium channels [52]. Thus, these are pivotal for neural cell interactions, brain plasticity and development [52, 72, 73]. We saw that PSA expression in the rodent striatum was very low (around 5%), as reported by Samal et al. [41]. However, upon LPS-injection, only the expression of one polysialylated structure was significantly increased, but in a very low abundance, indicating that it is not so much involved in neuroinflammatory signalling. A similar trend in upregulation of PSA-NCAM was seen in a Parkinsonian rat model (after injection of 6-hydroxydopamine to trigger neurodegeneration) as a marker for reactive astrocytes only at the site of lesion [74]. This corroborates the hypothesis that modulation of PSA's presence in the brain is associated with the reactivity, size and proliferation of resident cells and is upregulated in regions of high neuronal plasticity [75]. The increase in reactive astrocytes seen in our study at the injection site might be correlated with the increase seen in polysialylated structures.

Besides sialylation, fucosylation is another pivotal glycosylation trait in the brain [76] since it regulates neurite outgrowth and synaptic plasticity, being crucial for cognitive processes (reviewed by Schneider et al. [77]). This would be expected as an abundant distribution of fucosylated *N-*glycans was reported in the rodent brain [41], being higher than that in other mammalian tissues [76].

The cardinal role of core fucosylation in regulating neuronal functions is highlighted in a study by Fukuda et al., where mice lacking α1,6-fucosyltransferase (Fut8—an enzyme responsible for the attachment of a fucose residue to the core pentasaccharide characteristic of *N-*glycans) display behavioural impairments and schizophrenia-like behaviour [78]. This loss of Fut8 was later reported to decrease long-term potentiation in the hippocampus, impacting neuronal synaptic plasticity and, subsequently, learning and memory [79]. This accounts for the schizophrenia-like phenotype seen in the animals lacking this enzyme [79]. It has been reported that deficiency of α1,6-fucosyltransferase has a deleterious impact on glial cells (both astrocytes and microglia, in addition to the effect seen in neuronal cells), promoting their sensitivity to inflammatory mediators [48]. This might suggest that our LPS model (with an increase in reactive glial cells) impacts the production of Fut8, downregulating its expression, which contributes to a lower abundance of core fucosylated *N-*glycans. This feeds further into a positive loop that reinforces the neuroinflammatory cascades. However, further studies on this enzyme's expression will be required to confirm such hypothesis.

Regarding outer arm fucosylation, Kalovidouris et al. have shown that Fuc α1,2-Gal residues play a role in neuronal outgrowth and morphology, influencing long-term memory [80]. Additionally, outer arm fucosylation is also particularly important in the formation of LewisX epitopes (Galβ1-4 (Fucα1-3) GlcNAc) [81] and Sialyl-LewisX epitopes (LewisX epitope with a Neu5Ac residue attached to it), which are known to be central for brain development [82, 83]. A decrease in LewisX structures due to the absence of α1,3-fucosyltransferase IX (Fut9) was described to induce anxiety-like behaviour in mice [81, 84], and such behaviours have been reported in LPS models before [18]. The results seen in our LPS model come in accordance to this, since there is a decrease in the abundance of both *N-*glycans with LewisX epitopes and with Sialyl-LewisX epitopes, indicating that their absence might be contributing to the pathological condition, which might be both an effect as well as a cause of inflammatory cascades, participating in a cyclic chain of events.

Oligomannose structures are also essential players in neuroinflammatory processes due to the expression of mannose-binding lectins in astrocytes and microglia, which control immune responses through the lectin pathway of complement activation [85]. An increase in oligomannose *N-*glycans upregulates the binding to these receptors and, consequently, triggers the complement response, promoting further inflammation. This accords with our findings, since there is a significant increase in microglial activation/size and oligomannose structures, indicating the crosstalk between glycosylation and neuroinflammation. Nonetheless, additional studies on the expression of these lectins and other carbohydrate-binding proteins would be needed to confirm their influence in this condition.

The results seen through HILIC-UPLC were reinforced by MALDI MSI, with the added advantage of spatially characterising each glycan structure’s distribution. The expected increased expression of oligomannose and bisected glycans in the LPS-injected striatum was seen, as well as a decrease in fucosylated *N-*glycans in the overall hemisphere. Additionally, MALDI MSI allowed for the detection of specific structures that were not distinguished by HILIC-UPLC since only the major glycan constituent of each peak was considered for this latter analysis. This comes as a disadvantage in HILIC-UPLC since if each chromatogram peak corresponds to more than one glycan, then the ones in lower abundance will not be considered, resulting in an incomplete detection of some traits. Nevertheless, by combining it with *N-*glycans’ MALDI imaging, it is possible to have a complete characterisation of the *N-*glycome modulation upon neuroinflammation. This is of interest since it shows that *N-*glycans can be directly detected on tissue sections with high sensitivity and specificity.

It appears that there are specific structures that are dramatically increased upon inflammation, being characteristic of this event and promoted by it. In contrast, others seem to be significantly decreased in the whole hemisphere, suggesting a posterior effect of LPS/neuroinflammation at the *N-*glycome level and on such structures’ biosynthesis.

An aspect worth noting in this technique relates to the technical challenges in detecting sialic acids that were common during the past decades. Due to their instability, these can be easily lost in MSI steps, which decreases their detection [50, 51]. This can be circumvented by fragmentation of derivatised glycans to increase sialic acid stabilisation [50, 86, 87], which can be addressed by the new instrumentation’s advances. Nonetheless, following the results acquired by HILIC-UPLC, it seems that the abundance of sialic acids in the brain is overall reduced. Therefore, a low detection of sialic acid was expected. This low detection of sialic acids in the brain through MALDI imaging was also seen in a previous study where *N-*glycans from a mouse brain were analysed through MALDI quadrupole ion trap (QIT) time of flight (ToF) [88]. However, in that study, only 42 glycan structures were detected, whereas we detected 52 *N-*glycans in our study. This is most likely due to a newer generation of MALDI mass spectrometer that provides improved ionisation and higher resolution detection capabilities. MALDI imaging also allowed for the validation of the results quantified previously by HILIC-UPLC, emphasising the significant differences seen previously upon LPS-injection.

Overall, the quantifiable changes observed in individual *N-*glycans through both techniques (MALDI MSI and HILIC-UPLC) are suggestive for the role of both individual structures and general glycosylation traits in the neuroinflammatory cascade, which is worth exploring further.

Ideally, it would be of significant interest to assess the *N-*glycomic changes associated to each cell type (particularly with astrocytes and microglia) rather than in the whole tissue. However, the sensitivity and resolution of the technologies available do not allow for such detailed and specific analysis in brain tissue. For example, the resolution of 40 μm used in this study (while performing MALDI MSI) corresponds to approximately the same size of microglial cells (which are 40 μm to 50 μm in diameter in the rat cortex [89]), which does not allow for cell-specific *N*-glycome profiling.

Moreover, even though cell-specific *N-*glycosylation would potentially inform which cells are the major players in the glyco-dysregulations seen, the main goal of the study is to assess the overall *N*-glycomic changes, aiming to inform on a potential therapy to be administered in the whole striatum (and not just in specific cells). The relevance of looking at overall brain tissue *N*-glycosylation (not only in rodents but also in human clinical samples) has also been addressed in very recent reports by Gaunitz et al. [90] and Lee et al. [91], emphasising the interest and importance of such characterisation.

In summary, the combination of these multifaceted glyco-analytical techniques allowed for a detailed, quantifiable and spatial characterisation of the *N-*glycophenotype in a reproducible and comprehensively described model of neuroinflammation. This provides a strong starting point for similar approaches to be taken in preclinical models to decipher the undoubtedly crucial role of glycosylation in inflammatory brain injuries or diseases.

## Conclusions

This study presents the first step towards a deeper understanding of *N-*glycosylation’s role in an LPS-induced model of neuroinflammation. A significant decrease in sialylation and fucosylation was seen upon LPS-injection, suggesting that this downregulation might play a crucial role as a potentiator of inflammatory cascades. An overall increase in oligomannose and bisected *N-*glycan structures seen in the inflamed tissue could indicate their involvement in this phenomenon as well. The use of a combination of different glyco-analytical methodologies provides a complete knowledge of the brain’s glycomic profiling, which opens avenues for further research to be done on this field.

In the future, the use of in vivo models where pro-inflammatory stimuli/cytokines are employed will be even more relevant and of interest to dissect more precisely the mechanistic links between these and *N-*glycosylation phenomena in physiological conditions. Furthermore, potential studies focused on the regulation of glycosylation enzymes, on the expression of glycan-binding proteins (lectins) and on the glyco-profile of specific glycoproteins, will help to complete the molecular puzzle underlying this pathology and to further elucidate potential targets to be addressed in future therapies.

## Supplementary Information

**Additional file 1: Figure S1.** Confirmation of the neuroinflammatory effect of LPS on the rodent striatum by performing a negative control through injection of PBS into the striatum and analysis of the expression of neuroinflammatory markers in PBS-injected striatum vs the contralateral (non-injected (NI)) striatum at seven days post-injection (dpi). a. Experimental design of the study. Striatum of Sprague-Dawley rats was injected with PBS, and the animals were kept for seven days when tissues were collected for histological and transcriptomic analysis. b. Striatal mRNA expression of different genes related to inflammatory responses – Glial fibrillary protein (GFAP), Iba1 and Tumor necrosis factor α (TNFα). Results are expressed as means ± SEM. n=4-6; Mann-Whitney U test was used for between-group comparison, and statistically significant difference was set at **p<0.01. c., d., e. Histological evaluation of the expression of Iba1, GFAP and Vim (respectively) in PBS-injected vs NI striata at seven dpi. Scale bar = 50 μm. f., g., h. Striatal optical density of Iba+, GFAP+ or Vim+ (respectively) in the PBS-injected and NI striata. Results are expressed as means ± SEM. n=5-6. Paired Student t-test was used. NS=non-significant.

**Additional file 2: Figure S2.** *N-*glycan MALDI-MS spectra on the rat brain one week after LPS injection. Comparison of average mass spectra of (a) total *N-*glycans in the coronal brain section; (b) *N-*glycans detected in the LPS-injected striatum; (c) *N-*glycans detected in the non-injected (NI) striatum. Structures corresponding to the main significantly dysregulated GP in the HILIC-UPLC profile are described.

**Additional file 3: Figure S3.** *N-*glycome differences between LPS-injected and NI striata seen in MALDI-MSI to validate changes seen in HILIC-UPLC. a. MALDI-MSI spectra of *N*-glycans that were significantly differently expressed between LPS-injected and NI striata in UPLC. Next to each image is an intensity box plot that displays intensities of a given m/z interval filtered by the visible regions through their quartiles, specifically in the two analysed striata. The cloud part of the plot depicts how spectra of the striata are spread by intensity. Red dots refer to “outliers”. b. Summary table of the GP that are statistically significantly different between LPS-injected and NI striatum at seven dpi according to HILIC-UPLC and the respective intensity of distribution analysed through MALDI-MSI. Red indicates significantly increased peak area (abundance) in the LPS-injected striatum, whereas green represents significantly decreased peak area in the LPS-injected striatum, compared to NI striatum. The abundance of these was Log transformed for statistical analysis. N=5, paired Student's t-test was used to compare groups in each GP. ND – non-detected.

**Additional file 4: Figure S4.** Spatial distribution of sialylated and fucosylated *N-*glycans in the brain of LPS-injected rats. Frozen 10 μm thick coronal brain sections were subjected to MALDI-MSI and image spectra were acquired at a resolution of 40 μm. The panel shows representative individual sialylated and fucosylated *N-*glycan images and their distribution in the brain, allowing a spatial comparison between LPS-injected and non-injected (NI) striata. Each image is accompanied by the putative structures determined by combinations of accurate m/z, CID fragmentation patterns and glycan database structure. # refers to structures that were significantly differentially expressed in LPS-injected vs NI in HILIC-UPLC data.

**Additional file 5: Figure S5.** Spatial distribution of oligomannosylated, hybrid, bisected and neutral non-fucosylated *N-*glycans in the brain of LPS-injected rats. Frozen 10 μm thick coronal brain sections were subjected to MALDI-MSI and image spectra were acquired at a resolution of 40 μm. The panel shows representative individual neutral *N-*glycan images and their brain distribution, allowing a spatial comparison between LPS-injected and non-injected (NI) striata. Each image is accompanied by the putative structures determined by combinations of accurate m/z, CID fragmentation patterns and glycan database structure. # refers to structures that were significantly differentially expressed in LPS-injected vs non-injected in HILIC-UPLC data.

**Additional file 6: Figure S6.** Spatial distribution of putative individual *N-*glycans whose expression is altered upon LPS injection, at the injection site, at 200 μm and 400 μm from the injection site.

**Additional file 7: Table S1**. Composition of the rat striatum *N-*glycome categorised according to the main 26 glycan peaks, as per characterisation by Samal et al. in the HILIC-UPLC profile [41]. **Table S2.** Complete detailed MALDI-MSI data outlining m/z values and corresponding *N-*glycan structure as part of the spatial characterisation of the rat striatal *N*-glycome. **Table S3**. Calculation of derived glycosylation traits in the striatum.

## Data Availability

All data generated and analysed during this study are available from the corresponding author on reasonable request.

## Abbreviations

[18F]DPA714: [N,N-diethyl-2-(2-(4-(2-[18F]fluoroethoxy)phenyl)-5,7-dimethylpyrazolo[1, 5-a]pyrimidin-3-yl)acetamide]
BP_ND_: Binding potential
CNS: Central nervous system
dpi: Days post-injection
GFAP: Glial fibrillary acidic protein
HILIC-UPLC: Hydrophilic interaction liquid chromatography–ultra-performance liquid chromatography
LPS: Lipopolysaccharide
MALDI MSI: Matrix-assisted laser desorption ionisation mass spectrometry imaging
NI: Non-injected
PD: Parkinson’s disease
PET: Positron emission tomography
PSA: polysialic acid
TNF α: Tumour necrosis factor α
TSPO: 18 kD Translocator protein
Vim: Vimentin
