# Supplementary figures and images for: Complete spatial characterisation of N-glycosylation upon striatal neuroinflammation in the rodent brain
Source: J Neuroinflammation. 2021 May 16;18:116. doi: 10.1186/s12974-021-02163-6 (PMC8127229; doi:10.1186/s12974-021-02163-6)

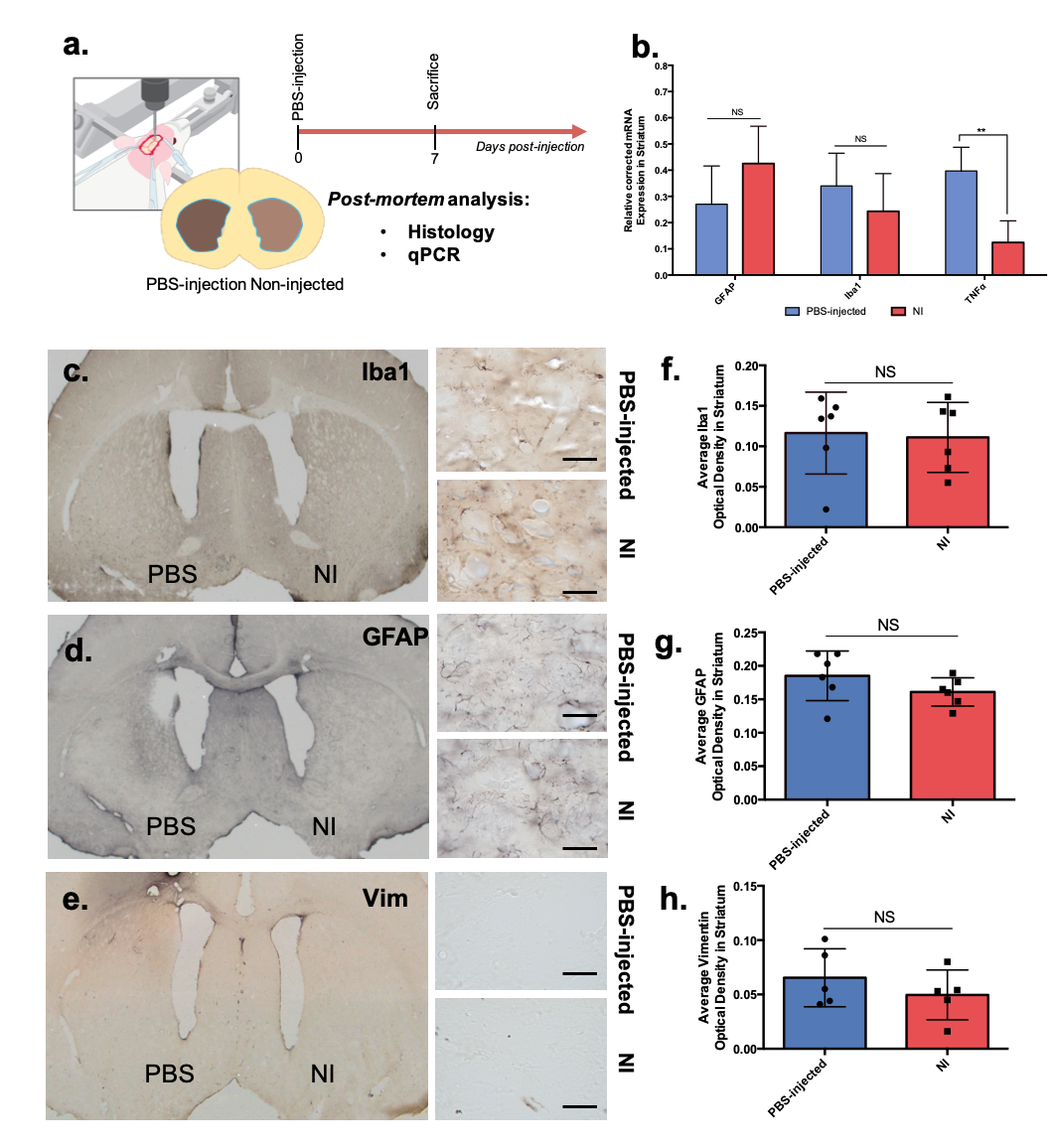

Supplement: Supplementary file 1 — Additional file 1: Figure S1. Confirmation of the neuroinflammatory effect of LPS on the rodent striatum by performing a negative control through injection of PBS into the striatum and analysis of the expression of neuroinflammatory markers in PBS-injected striatum vs the contralateral (non-injected (NI)) striatum at seven days post-injection (dpi). a. Experimental design of the study. Striatum of Sprague-Dawley rats was injected with PBS, and the animals were kept for seven days when tissues were collected for histological and transcriptomic analysis. b. Striatal mRNA expression of different genes related to inflammatory responses – Glial fibrillary protein (GFAP), Iba1 and Tumor necrosis factor α (TNFα). Results are expressed as means ± SEM. n=4-6; Mann-Whitney U test was used for between-group comparison, and statistically significant difference was set at **p<0.01. c., d., e. Histological evaluation of the expression of Iba1, GFAP and Vim (respectively) in PBS-injected vs NI striata at seven dpi. Scale bar = 50 μm. f., g., h. Striatal optical density of Iba+, GFAP+ or Vim+ (respectively) in the PBS-injected and NI striata. Results are expressed as means ± SEM. n=5-6. Paired Student t-test was used. NS=non-significant. [file 12974_2021_2163_MOESM1_ESM.png]

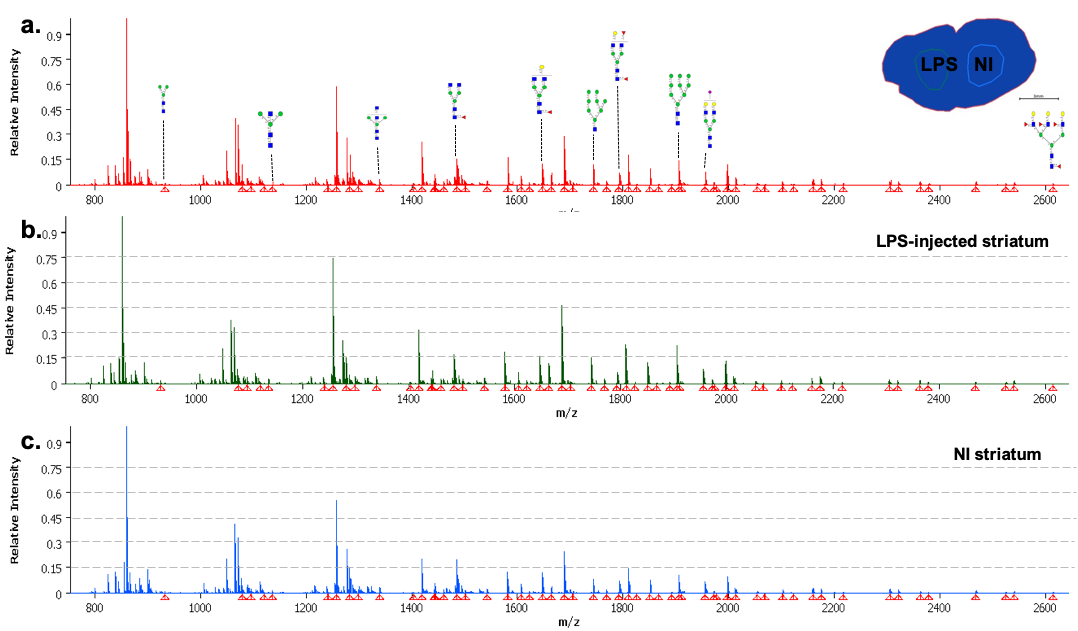

Supplement: Supplementary file 2 — Additional file 2: Figure S2. N-glycan MALDI-MS spectra on the rat brain one week after LPS injection. Comparison of average mass spectra of (a) total N-glycans in the coronal brain section; (b) N-glycans detected in the LPS-injected striatum; (c) N-glycans detected in the non-injected (NI) striatum. Structures corresponding to the main significantly dysregulated GP in the HILIC-UPLC profile are described. [file 12974_2021_2163_MOESM2_ESM.png]

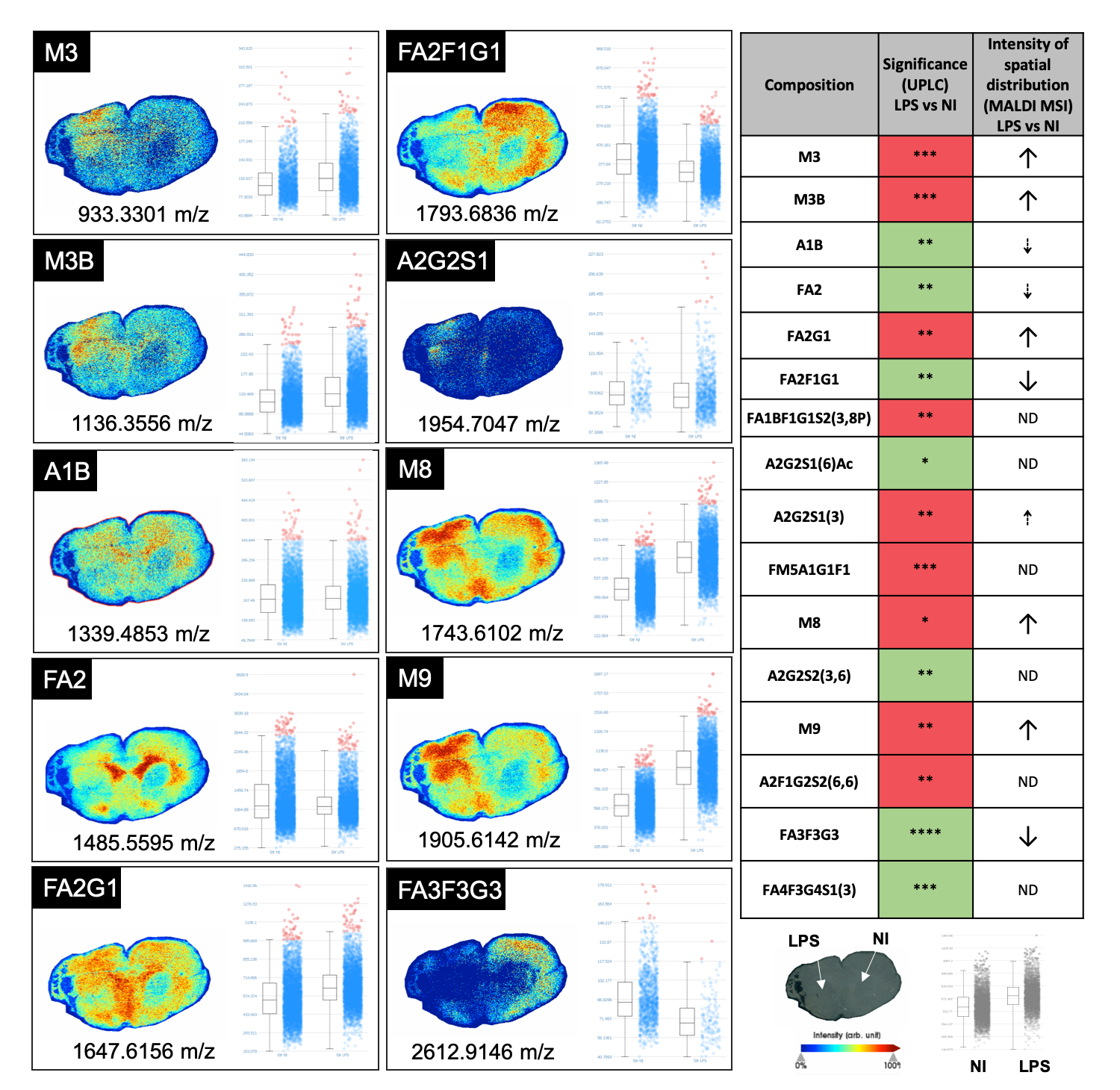

Supplement: Supplementary file 3 — Additional file 3: Figure S3. N-glycome differences between LPS-injected and NI striata seen in MALDI-MSI to validate changes seen in HILIC-UPLC. a. MALDI-MSI spectra of N-glycans that were significantly differently expressed between LPS-injected and NI striata in UPLC. Next to each image is an intensity box plot that displays intensities of a given m/z interval filtered by the visible regions through their quartiles, specifically in the two analysed striata. The cloud part of the plot depicts how spectra of the striata are spread by intensity. Red dots refer to “outliers”. b. Summary table of the GP that are statistically significantly different between LPS-injected and NI striatum at seven dpi according to HILIC-UPLC and the respective intensity of distribution analysed through MALDI-MSI. Red indicates significantly increased peak area (abundance) in the LPS-injected striatum, whereas green represents significantly decreased peak area in the LPS-injected striatum, compared to NI striatum. The abundance of these was Log transformed for statistical analysis. N=5, paired Student's t-test was used to compare groups in each GP. ND – non-detected. [file 12974_2021_2163_MOESM3_ESM.png]

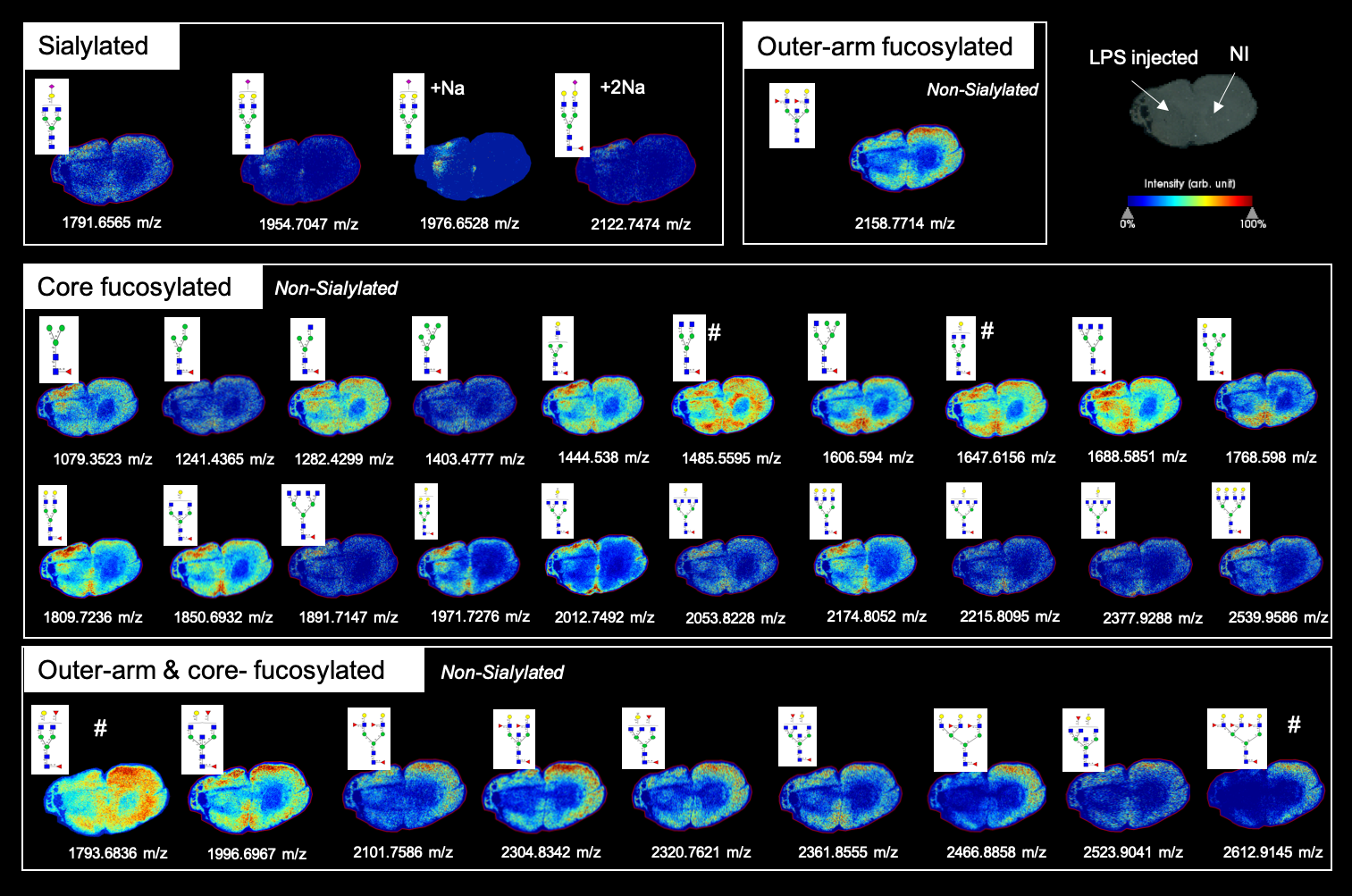

Supplement: Supplementary file 4 — Additional file 4: Figure S4. Spatial distribution of sialylated and fucosylated N-glycans in the brain of LPS-injected rats. Frozen 10 μm thick coronal brain sections were subjected to MALDI-MSI and image spectra were acquired at a resolution of 40 μm. The panel shows representative individual sialylated and fucosylated N-glycan images and their distribution in the brain, allowing a spatial comparison between LPS-injected and non-injected (NI) striata. Each image is accompanied by the putative structures determined by combinations of accurate m/z, CID fragmentation patterns and glycan database structure. # refers to structures that were significantly differentially expressed in LPS-injected vs NI in HILIC-UPLC data. [file 12974_2021_2163_MOESM4_ESM.png]

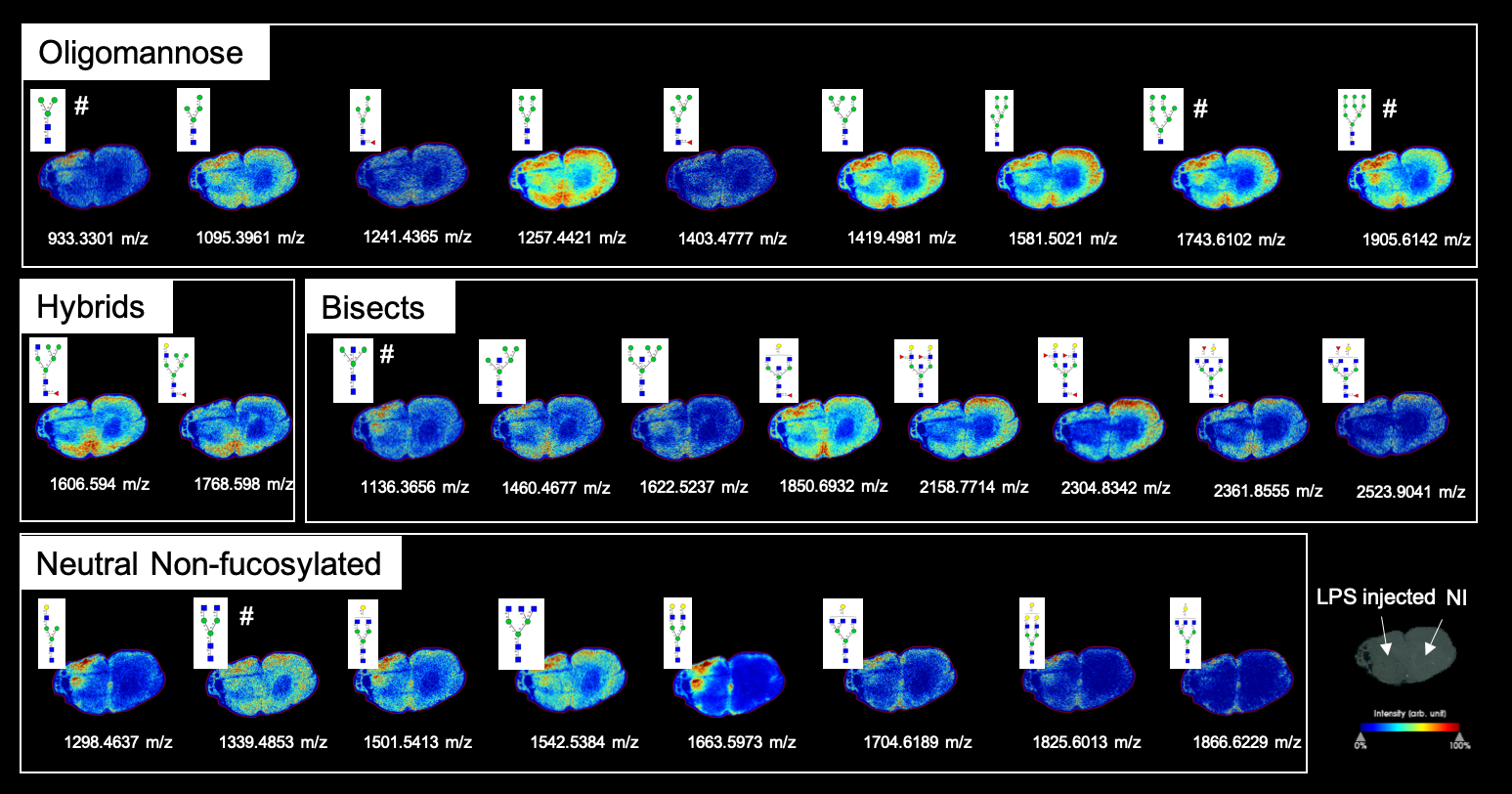

Supplement: Supplementary file 5 — Additional file 5: Figure S5. Spatial distribution of oligomannosylated, hybrid, bisected and neutral non-fucosylated N-glycans in the brain of LPS-injected rats. Frozen 10 μm thick coronal brain sections were subjected to MALDI-MSI and image spectra were acquired at a resolution of 40 μm. The panel shows representative individual neutral N-glycan images and their brain distribution, allowing a spatial comparison between LPS-injected and non-injected (NI) striata. Each image is accompanied by the putative structures determined by combinations of accurate m/z, CID fragmentation patterns and glycan database structure. # refers to structures that were significantly differentially expressed in LPS-injected vs non-injected in HILIC-UPLC data. [file 12974_2021_2163_MOESM5_ESM.png]

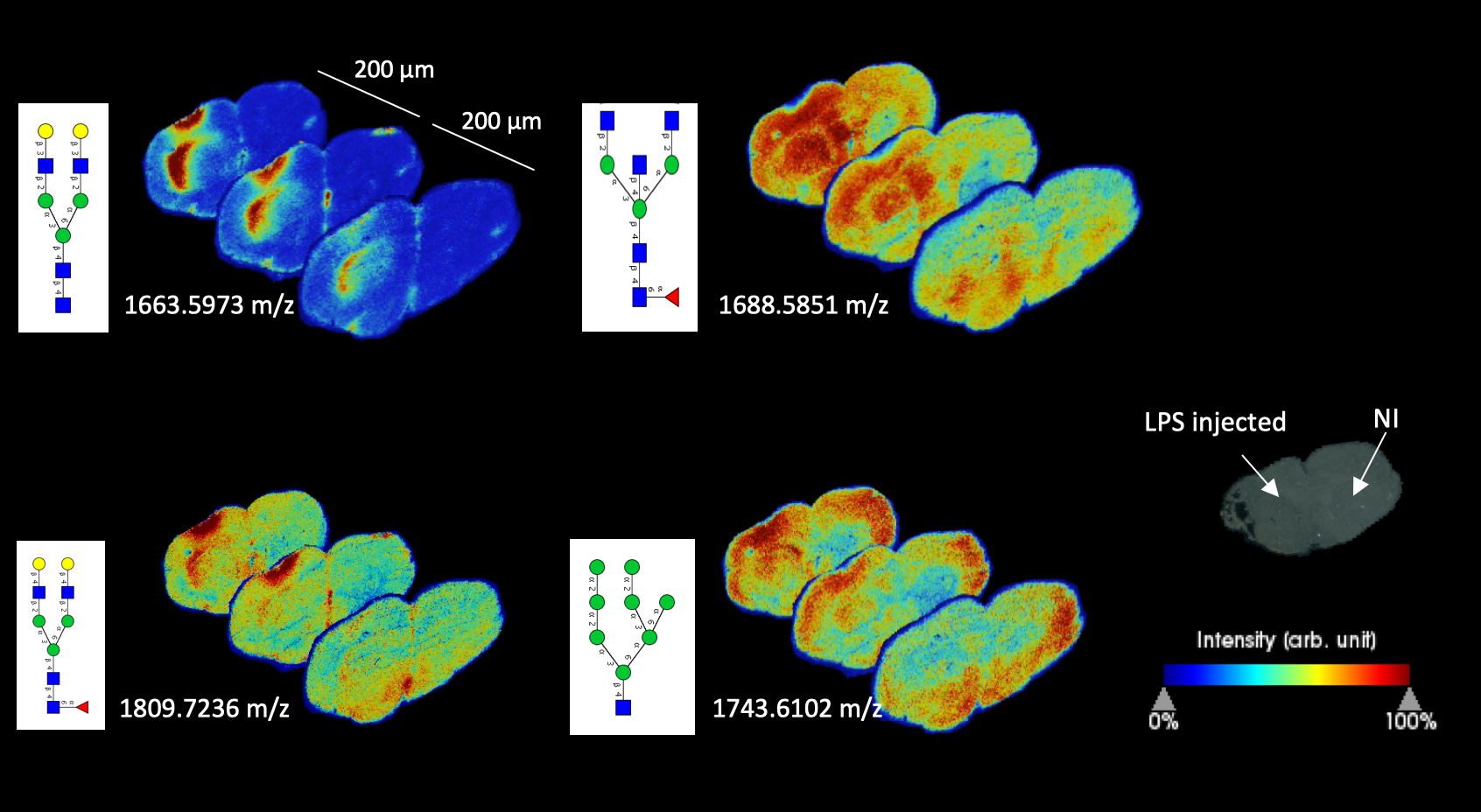

Supplement: Supplementary file 6 — Additional file 6: Figure S6. Spatial distribution of putative individual N-glycans whose expression is altered upon LPS injection, at the injection site, at 200 μm and 400 μm from the injection site. [file 12974_2021_2163_MOESM6_ESM.png]
