# Supplementary material for: Complete spatial characterisation of N-glycosylation upon striatal neuroinflammation in the rodent brain
Source: J Neuroinflammation. 2021 May 16;18:116. doi: 10.1186/s12974-021-02163-6 (PMC8127229; doi:10.1186/s12974-021-02163-6)
Supplement: Supplementary file 7 — Additional file 7: Table S1. Composition of the rat striatum N-glycome categorised according to the main 26 glycan peaks, as per characterisation by Samal et al. in the HILIC-UPLC profile [41]. Table S2. Complete detailed MALDI-MSI data outlining m/z values and corresponding N-glycan structure as part of the spatial characterisation of the rat striatal N-glycome. Table S3. Calculation of derived glycosylation traits in the striatum. [file 12974_2021_2163_MOESM7_ESM.docx]

**Supplementary Tables**

**Table S1. Composition of the rat striatum *N-*glycome categorised according to the main 26 glycan peaks, as per characterisation by Samal et al. in the HILIC-UPLC profile** ^41^. A more detailed characterisation can be found at this reference. These are the significant glycans' structures identified in each chromatographic peak and the average percentage area for each peak in LPS-injected striatum vs non-injected (NI) striatum. Structural schemes are given in accordance with Symbol Nomenclature for Glycan (SNFG) format terms of *N*-Acetylglucosamine (
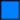
), mannose (
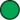
), fucose (
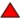
), galactose (
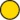
) and sialic acid (Neu5Ac
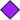
 and Neu5Gc
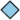
). Statistical significance between groups is also described, as well as the p-values derived from a paired Student t-test comparison of glycan peaks between different groups after Log transforming each GP area.

|  | **Composition^#^** | **Structure^#^** | **Average peak area (%) in LPS-injected striatum** | **Average peak area (%) in NI striatum** | **Significance** | ***p-value*** |
| --- | --- | --- | --- | --- | --- | --- |
| **GP1** | M3 | 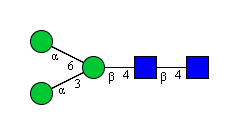 | 0.368±0.078 | 0.098±0.025 | *** | 0.0009 |
| **GP2** | M3B | 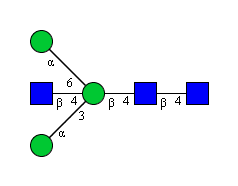 | 1.884±0.183 | 1.116±0.051 | *** | 0.0004 |
| **GP3** | A1B | 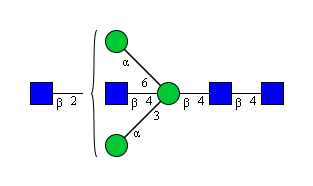 | 0.724±0.050 | 0.802±0.042 | ** | 0.0050 |
| **GP4** | FA2 | 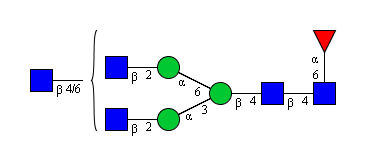 | 4.03±0.180 | 5.128±0.188 | ** | 0.0018 |
| **GP5** | FA3 | 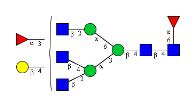 | 20.3±0.621 | 20.81±0.986 | ns | 0.4326 |
| **GP6** | FA2G1 | 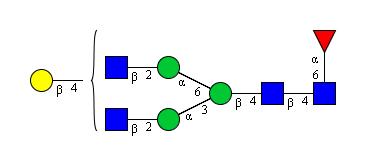 | 1.346±0.044 | 1.086±0.077 | ** | 0.0020 |
| **GP7** | A2F1G1 | 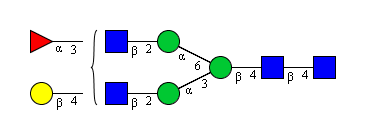 | 0.17±0.025 | 0.186±0.018 | ns | 0.3977 |
| **GP8** | M6 | 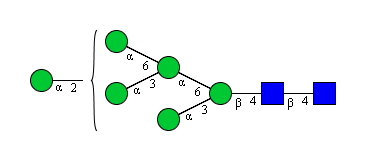 | 8.056±0.226 | 7.896±0.149 | ns | 0.3091 |
| **GP9** | FA2F1G1 | 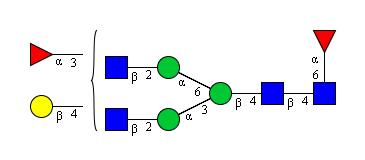 | 2.078±0.150 | 2.958±0.114 | ** | 0.0012 |
| **GP10** | FA1BF1G1S(3,8P)2 | 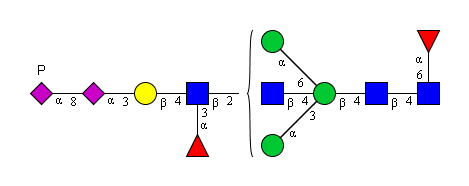 | 2.12±0.075 | 1.842±0.056 | ** | 0.0017 |
| **GP11** | FA3F1G1 | 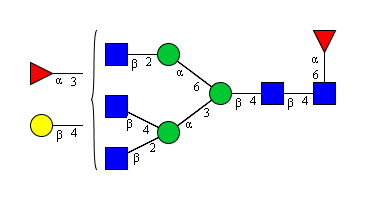 | 2.94±0.286 | 3.334±0.198 | ns | 0.0891 |
| **GP12** | A2G2S(6Ac)1 | 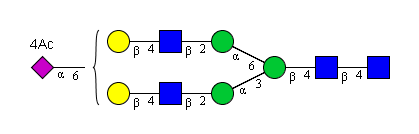 | 1.588±0.106 | 1.886±0.091 | * | 0.0161 |
| **GP13** | M7D1 | 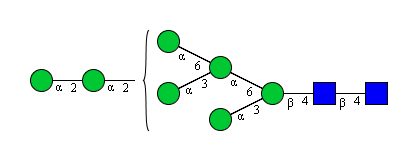 | 6.564±0.113 | 6.526±0.165 | ns | 0.7319 |
| **GP14** | A2G2S(3)1 | 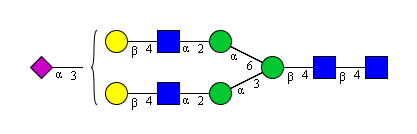 | 2.21±0.064 | 2.006±0.046 | ** | 0.0036 |
| **GP15** | FM5A1F1G1 | 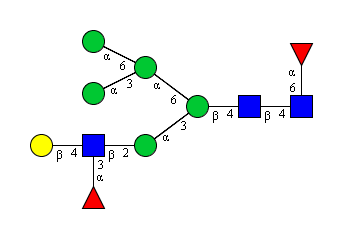 | 4.108±0.101 | 3.164±0.137 | *** | 0.0004 |
| **GP16** | M8 | 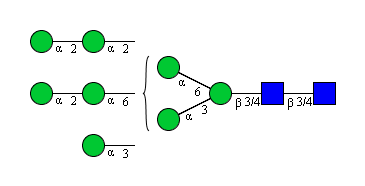 | 4.884±0.202 | 4.394±0.151 | * | 0.0200 |
| **GP17** | FA2F2G2 | 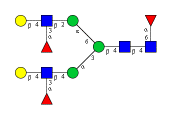 | 4.602±0.064 | 4.288±0.339 | ns | 0.1517 |
| **GP18** | FA3F2G2 | 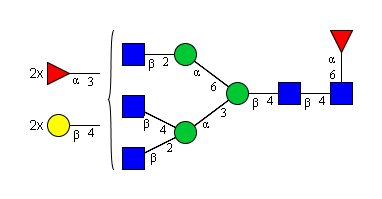 | 1.564±0.073 | 1.66±0.083 | ns | 0.1678 |
| **GP19** | A2G2S(3,6)2 | 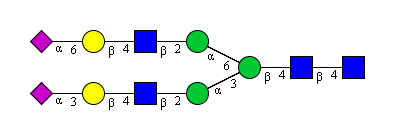 | 0.668±0.088 | 0.9±0.032 | ** | 0.0057 |
| **GP20** | M9 | 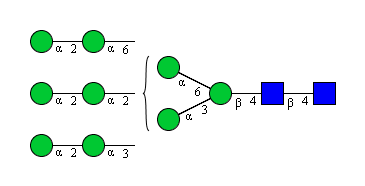 | 11.898±0.722 | 9.598±0.211 | ** | 0.0016 |
| **GP21** | A2F1G2S(6,6)2 | 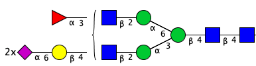 | 2.908±0.147 | 2.488±0.061 | ** | 0.0063 |
| **GP22** | FA3F2G3S(3)1 | 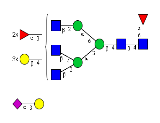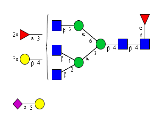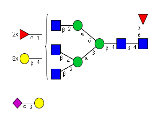 | 4.57±0.157 | 4.764±0.242 | ns | 0.1836 |
| **GP23** | FA3F3G3 | 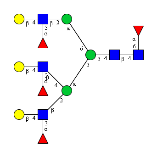 | 3.262±0.092 | 4±0.132 | **** | <0.0001 |
| **GP24** | FA3F1G3S(3,3,3)3 |  | 2.29±0.123 | 2.212±0.105 | ns | 0.2463 |
| **GP25** | FA4F3G4S(3)1 | 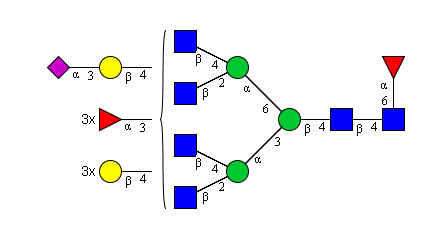 | 2.45±0.156 | 4.154±0.383 | *** | 0.0001 |
| **GP26** | FA4F2G4S(3,8P)2 | 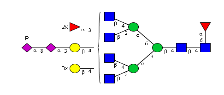 | 2.424±0.252 | 2.706±0.277 | ns | 0.1995 |

**Table S2. Complete detailed MALDI-MSI data outlining m/z values and corresponding *N-*glycan structure as part of the spatial characterisation of the rat striatal *N*-glycome.** Structural schemes are given in accordance with Symbol Nomenclature for Glycan (SNFG) format terms of *N*-Acetylglucosamine (
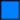
), mannose (
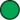
 ), fucose (
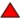
), galactose (
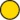
) and sialic acid (Neu5Ac
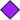
 and Neu5Gc
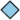
)).

| Observed [m/z]  [M+Na]+ | Monosaccharide composition | Structure |
| --- | --- | --- |
| 933.3301 m/z | Hex3HexNAc2 | 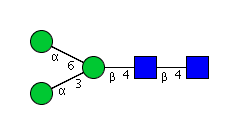 |
| 1079.3523 m/z | Hex3dHex1HexNAc2 | 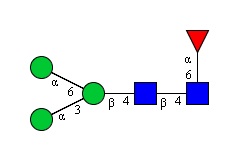 |
| 1095.3961 m/z | Hex4HexNAc2 | 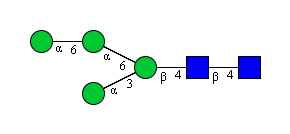 |
| 1136.3656 m/z | Hex3HexNAc3 | 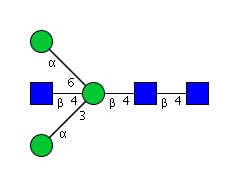 |
| 1241.4365 m/z | Hex4dHex1HexNAc2 | 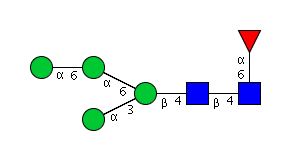 |
| 1257.4421 m/z | Hex5HexNAc2 | 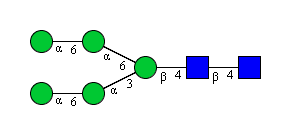 |
| 1282.4299 m/z | Hex3dHex1HexNAc3 | 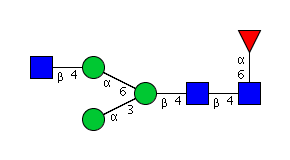 |
| 1298.4637 m/z | Hex4HexNAc3 | 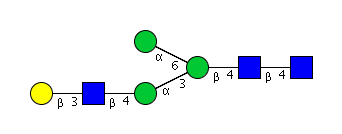 |
| 1339.4853m/z | Hex3HexNAc4 | 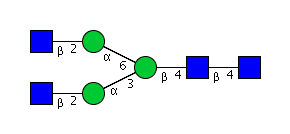 |
| 1403.4777m/z | Hex5dHex1HexNAc2 | 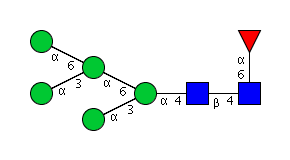 |
| 1419.4981 m/z | Hex6HexNAc2 | 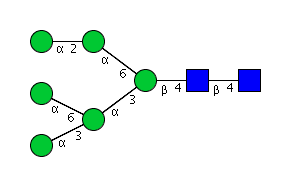 |
| 1444.5380 m/z | Hex4dHex1HexNAc3 | 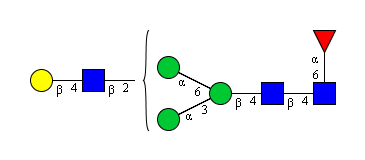 |
| 1460.4677 m/z | Hex5HexNAc3 | 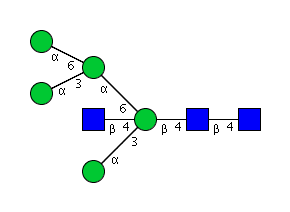 |
| 1485.5595 m/z | Hex3dHex1HexNAc4 | 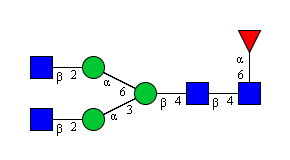 |
| 1501.5413 m/z | Hex4HexNAc4 | 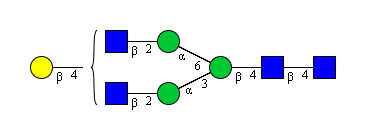 |
| 1542.5384 m/z | Hex3HexNAc5 | 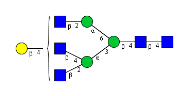 |
| 1581.5021 m/z | Hex7HexNAc2 | 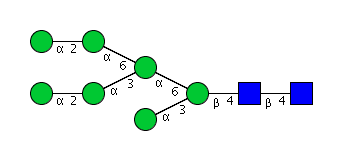 |
| 1606.594 m/z | Hex5dHex1HexNAc3 | 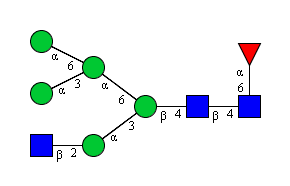 |
| 1622.5237 m/z | Hex6HexNAc3 | 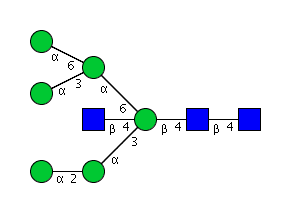 |
| 1647.6156 m/z | Hex4dHex1HexNAc4 | 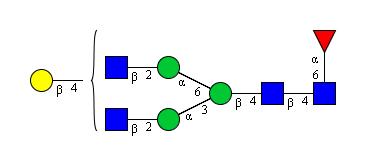 |
| 1663.5973 m/z | Hex5HexNAc4 | 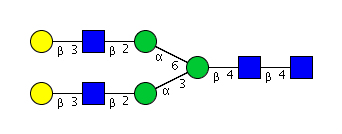 |
| 1688.5851 m/z ± | Hex3dHex1HexNAc5 | 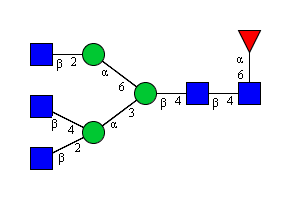 |
| 1704.6189 m/z | Hex4HexNAc5 | 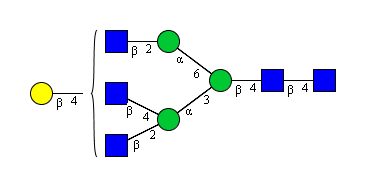 |
| 1743.6102 m/z | Hex8HexNAc2 | 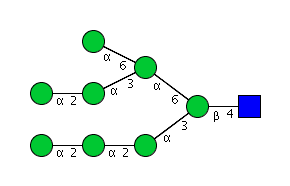 |
| 1768.598 m/z | Hex6dHex1HexNAc3 | 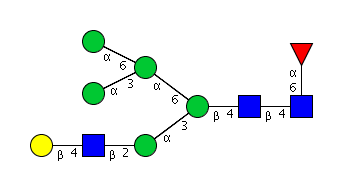 |
| 1791.6565 m/z | Hex4HexNAc4NeuAc1 | 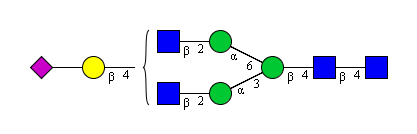 |
| 1793.6836 m/z | Hex4dHex2HexNAc4 | 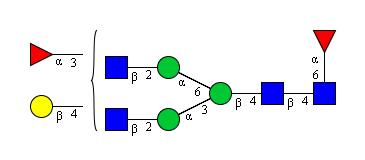 |
| 1809.7236 m/z | Hex5dHex1HexNAc4 | 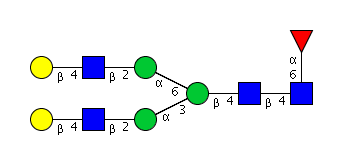 |
| 1825.6013 m/z | Hex6HexNAc4 | 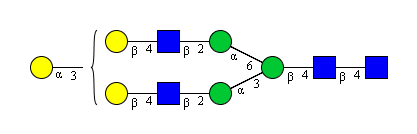 |
| 1850.6932 m/z | Hex4dHex1HexNAc5 | 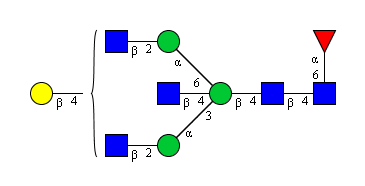 |
| 1866.6229 m/z | Hex5HexNAc5 | 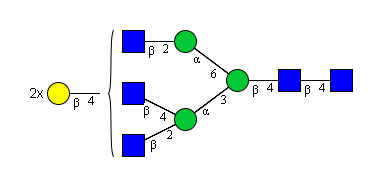 |
| 1891.7147 m/z | Hex3dHex1HexNAc6 | 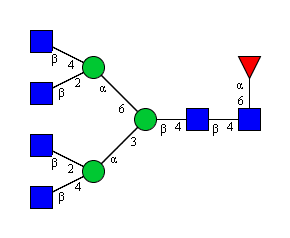 |
| 1905.6142 m/z | Hex9HexNAc2 | 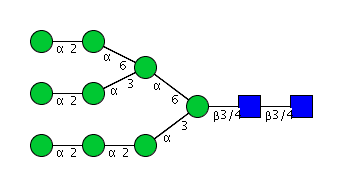 |
| 1954.7047 m/z | Hex5HexNAc4NeuAc1 | 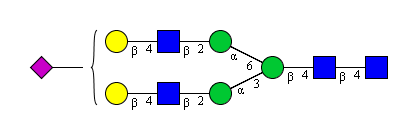 |
| 1971.7276 m/z | Hex6dHex1HexNAc4 | 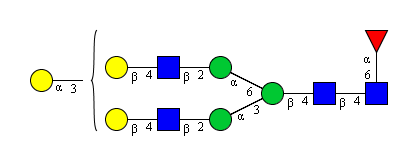 |
| 1996.6967 m/z | Hex4dHex2HexNAc5 | 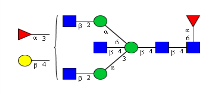 |
| 2012.7492 m/z | Hex5dHex1HexNAc5 | 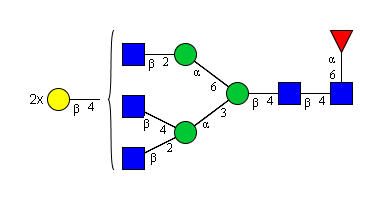 |
| 2053.8228 m/z | Hex4dHex1HexNAc6 | 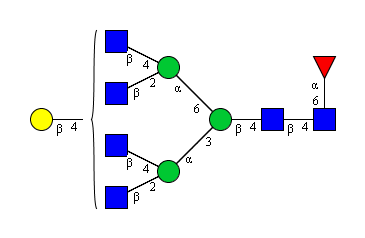 |
| 2067.7223 m/z | Hex10HexNAc2 | 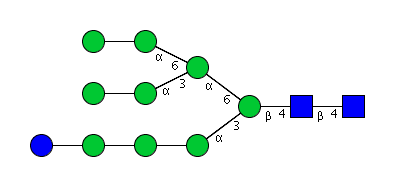 |
| 2101.7586 m/z | Hex5dHex3HexNAc4 | 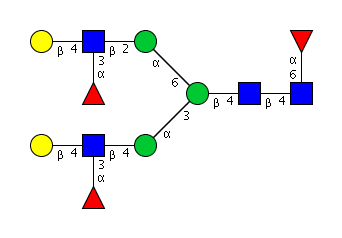 |
| 2122.7474 m/z | Hex5dHex1HexNAc4NeuAc1 + 2Na | 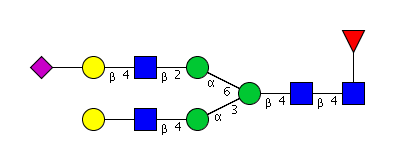 |
| 2158.7714 m/z | Hex5dHex2HexNAc5 | 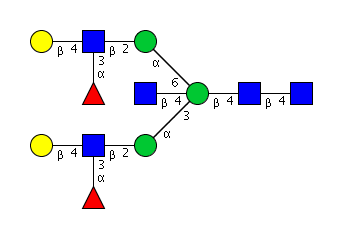 |
| 2174.8052 m/z | Hex6dHex1HexNAc5 | 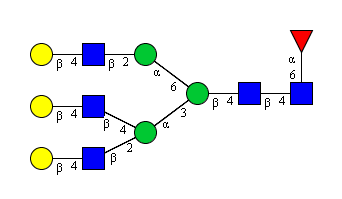 |
| 2215.8095 m/ | Hex5dHex1HexNAc6 | 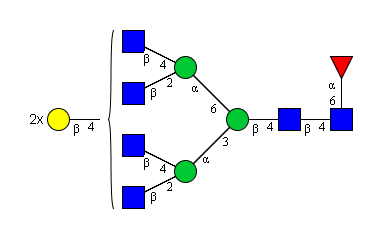 |
| 2304.8342 m/z | Hex5dHex3HexNAc5 | 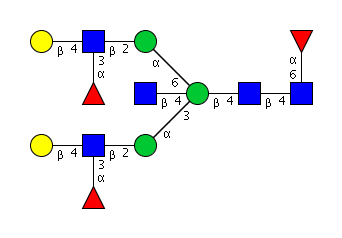 |
| 2320.7621 m/z | Hex6dHex2HexNAc5 | 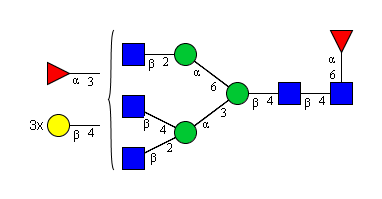 |
| 2361.8555 m/z | Hex5dHex2HexNAc6 | 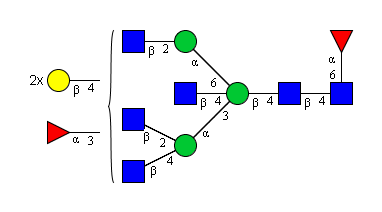 |
| 2377.9288 m/z | Hex6dHex1HexNAc6 | 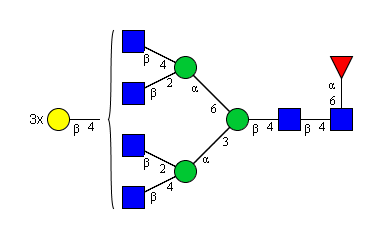 |
| 2466.8858 m/z | Hex6dHex3HexNAc5 | 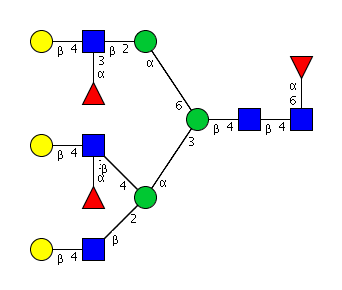 |
| 2523.9041 m/z | Hex6dHex2HexNAc6 | 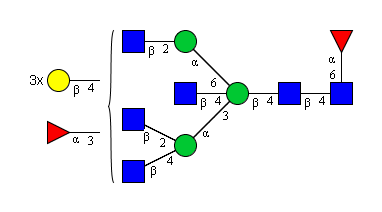 |
| 2539.9586 m/z | Hex7dHex1HexNAc6 | 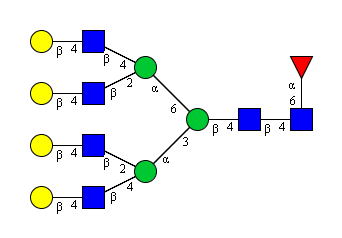 |
| 2612.9145 m/z | Hex6dHex4HexNAc5 | 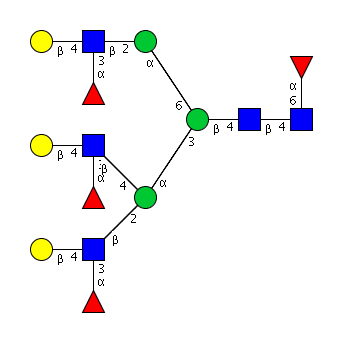 |

**Table S3. Calculation of derived glycosylation traits in the striatum.** The description of each glycosylation trait and the calculation rationale are presented as follows.

| **Derived Traits** | **Charge analysis** | N1 | The percentage of total neutral glycans in total glycans | SUM(GP1:9,GP11,GP13,GP15:GP18, GP20, GP23) |
| --- | --- | --- | --- | --- |
|  |  | S^Total^ | The percentage of total sialylated glycans in total glycans | SUM(GP10, GP12,GP14,GP19,GP21,GP22,GP24:26) |
|  |  | S1 | The percentage of mono-sialylated glycans in total glycans | SUM(GP14, GP25, GP22) |
|  |  | S2 | The percentage of di-sialylated glycans in total glycans | SUM(GP10, GP19, GP21, GP26) |
|  |  | S3 | The percentage of tri-sialylated glycans in total glycans | GP24 |
|  |  | Phos | The percentage of phosphorylated glycans in total glycans | GP22 |
|  |  | S^Ac^ | The percentage of acetylated charged species | GP12 |
|  |  | Total charged species | The percentage of total charged species in total glycans | SUM(GP10, GP12,GP14,GP19,GP21,GP22,GP24:26) |
|  |  | Poly | The percentage of polysialic acids in total glycans | SUM (GP10, GP26) |
|  | **Branching** | A1 | The percentage of monoantennary glycans in total glycans | GP15 |
|  |  | A2 or A1B | The percentage of biantennary (or bisecting monoantennary) glycans in total glycans | SUM(GP3,GP4,GP6,GP7,GP9, GP10, GP12, GP14, GP17, GP19, GP21) |
|  |  | A3 or A2B | The percentage of triantennary (or bisecting diantennary) glycans in total glycans | SUM(GP5, GP11, GP18, GP22, GP23, GP24) |
|  |  | A4 or A3B | The percentage of tetraantennary (or bisecting triantennary) glycans in total glycans | SUM(GP25, GP26) |
|  | **Oligomannose** | Total oligomannose | The percentage of oligomannose structures in total glycans | SUM(GP1,GP2, GP8, GP13, GP16, GP20) |
|  |  | Lower order oligomannose | The percentage of oligomannose structures from M1-M5 in total glycans | SUM(GP1, GP2) |
|  |  | Higher order oligomannose | The percentage of oligomannose structures from M6-M9 in total glycans | SUM(GP8, GP13, GP16, GP20) |
|  | **Hybrid** | Total hybrid glycans | The percentage of hybrid structures in total glycans | SUM (GP15) |
|  | **Core fucosylation** | CoreF^Total^ | The percentage of core fucosylated glycans in total glycans | SUM(GP4: GP6, GP9: GP11, GP15, GP17, GP18, GP22:GP26) |
|  | **Outer arm fucosylation** | Outer armF^Total^ | The percentage of outer arm fucosylated glycans in total glycans | SUM(GP7, GP9, GP10, GP11, GP15, GP17, GP18, GP21:GP26) |
|  |  | Lewis^X^ epitopes | The percentage of glycans containing Lewis^X^ epitopes in total glycans | SUM(GP7, GP9, GP11, GP15, GP17, GP18, GP22:GP23, GP25, GP26) |
|  |  | Sialyl-Lewis^X^ epitopes | The percentage of glycans containing Sialyl-Lewis^X^ epitopes in total glycans | SUM(GP10, GP21, GP22, GP24, GP25, GP26) |
|  | **Total fucose residues** | F1 | The percentage of mono-fucosylated glycans in total glycans | SUM(GP7, GP9, GP10, GP11, GP15, GP17, GP18, GP21:GP26) |
|  |  | F2 | The percentage of di-fucosylated glycans in total glycans | SUM(GP9,GP10,GP11,GP15,GP24) |
|  |  | F3 | The percentage of tri-fucosylated glycans in total glycans | SUM(GP17,GP18,GP22,GP26) |
|  |  | F4 | The percentage of tetra-fucosylated glycans in total glycans | SUM(GP23,GP25) |
|  | **Bisected** | Bisected | The percentage of bisected glycans in total glycans | SUM (GP2, GP3, GP10) |
